# Identifying the “demon whale-biter”: Patterns of scarring on large whales attributed to a cookie-cutter shark *Isistius* sp

**DOI:** 10.1371/journal.pone.0152643

**Published:** 2016-04-07

**Authors:** Peter B. Best, Theoni Photopoulou

**Affiliations:** 1 Mammal Research Institute, Department of Zoology and Entomology, University of Pretoria, Pretoria, South Africa; 2 Centre for Statistics in Ecology, Environment and Conservation, Department of Statistical Sciences, University of Cape Town, Rondebosch, South Africa; University of St Andrews, UNITED KINGDOM

## Abstract

The presence of crater-like wounds on cetaceans and other large marine vertebrates and invertebrates has been attributed to various organisms. We review the evidence for the identity of the biting agent responsible for crater wounds on large whales, using data collected from sei (*Balaenoptera borealis*), fin (*B*. *physalus*), inshore and offshore Bryde’s (*B*. *brydeii* sp) and sperm whales (*Physeter macrocephalus*) examined at the Donkergat whaling station, Saldanha Bay, South Africa between March and October 1963. We then analyse the intensity and trends in its predation on large whales. Despite the scarcity of local records, we conclude that a cookie-cutter shark *Isistius* sp is the most likely candidate. We make inferences about the trends in (1) total counts of unhealed bitemarks, and (2) the proportion of unhealed bitemarks that were recent. We use day of the year; reproductive class, social grouping or sex; depth interval and body length as candidate covariates. The models with highest support for total counts of unhealed bitemarks involve the day of the year in all species. Depth was an important predictor in all species except offshore Bryde’s whales. Models for the proportion of recent bites were only informative for sei and fin whales. We conclude that temporal scarring patterns support what is currently hypothesized about the distribution and movements of these whale species, given that *Isistius* does not occur in the Antarctic and has an oceanic habitat. The incidence of fresh bites confirms the presence of *Isistius* in the region. The lower numbers of unhealed bites on medium-sized sperm whales suggests that this group spends more time outside the area in which bites are incurred, providing a clue to one of the biggest gaps in our understanding of the movements of mature and maturing sperm males.

## Background

**Fig 1:**
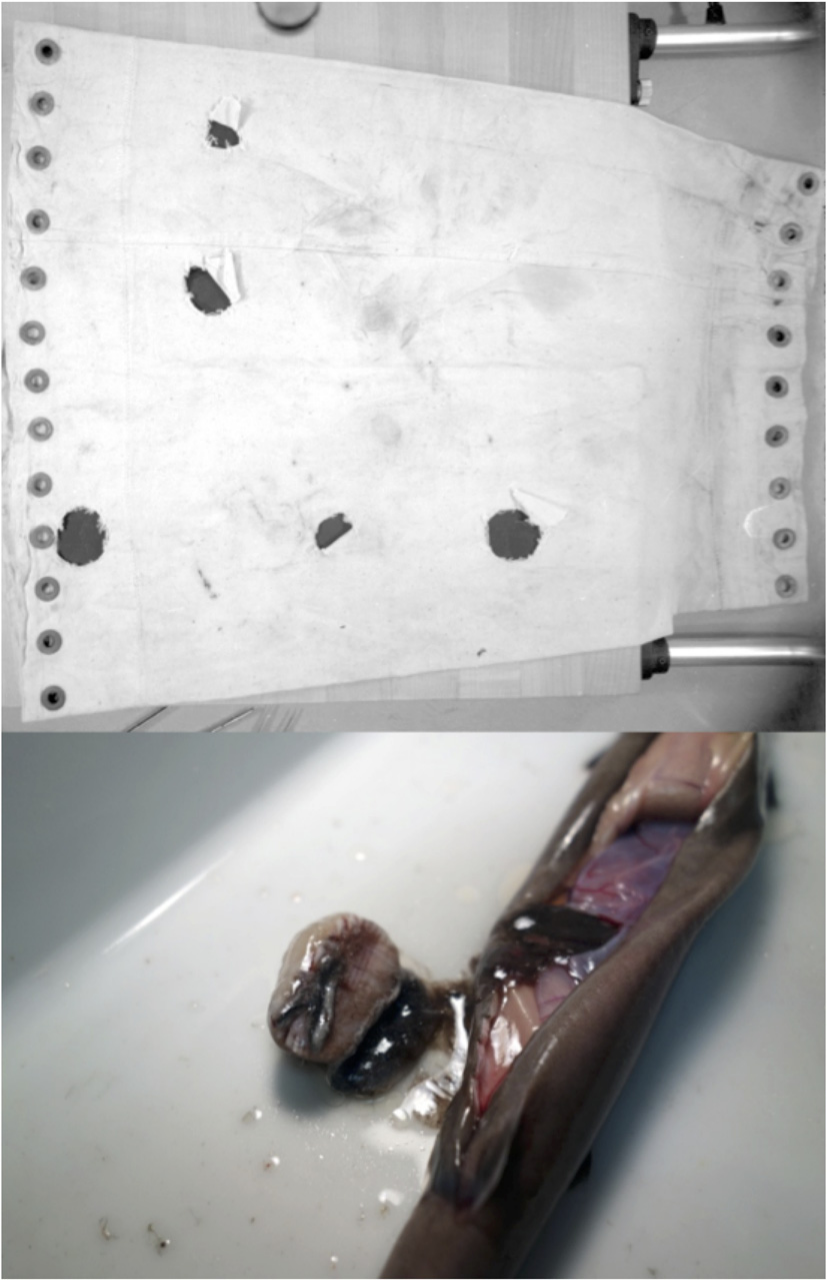
(above) Cod end of an Isaacs-Kidd midwater trawl bitten by captured Isistius brasiliensis (photo J C Staiger, University of Miami, 10 May 1972); (below) plug of fish tissue found in the stomach of an *I*. *brasiliensis* caught in Isaacs-Kidd midwater trawl (photo M J Miller, Atmosphere and Ocean Research Institute, University of Tokyo, Japan).

The presence of crater-like wounds on cetaceans and other large vertebrate and invertebrate marine organisms puzzled observers for many years, and was variously attributed to the physical displacement of *Coronula* barnacles [1], attachments of the copepod parasite *Pennella* [2,3], bites of sucking fish including lampreys [2,4], lamellae on the discs of remoras [5] and even infection by microorganisms [6]. Subsequently the most likely candidates have been identified as small pelagic sharks of the genus *Isistius* [7,8]. These are too small (up to about 500mm total length) to be regularly taken by fisheries although they are occasionally caught by pelagic longlines, and sometimes in midwater trawls and plankton nets (Fig 1). Consequently knowledge of their distribution and behavior is patchy, and the evidence of their involvement as the responsible biting agent is largely circumstantial.

Only the distribution of the commonest species, *Isistius brasiliensis*, is known with any confidence: it appears to have a circumpolar distribution in tropical waters, with records in latitudes greater than 40°N and S being rare and sometimes erroneous [9]. The southernmost record appears to be an observed take in a stern trawl at 41°40’ S, near Tasmania [10]. If this is the biting agent, its absence from higher latitudes of the Southern Hemisphere would be consistent with the observations that large whales in the Antarctic between October and the following May generally lacked the open unhealed “pits” found on whales in South African waters [6,11,12] and only rarely were partly healed “pits” to be found [6]. This was used as early de facto evidence of seasonal migration between lower and higher latitudes in southern baleen whales [6]. Latterly, differences in degrees of such scarring have also been used to discriminate between cetacean populations [13–16] and even individuals [17,18]. With the exception of [14], however, no attempts have been made to quantify the total level of scarring in cetaceans, presumably because in the living animal only a portion of the body is visible above the surface at any one time (and rarely the ventrum where much of the scarring occurs). The lack of quantitative analyses of trends in scarring has limited our knowledge of the intensity of predation by the biting agent and how it might vary regionally and seasonally, both among and within prey types.

In this paper we provide a review the evidence for the identity of the biting agent (S1 Text), and then analyse data on the incidence of fresh crater wounds and the relative degree of scarring from old bites on whales landed at Donkergat, South Africa, in 1963. At a latitude of 33° S, this station was strategically placed to sample both northern and southern migrations of whales, over a baleen whaling season lasting 6 months (that in 1963 was effectively extended to 8 months with the issuing by the South African Government of a permit under Article VIII of the International Convention for the Regulation of Whaling to take up to 50 sei/Bryde’s whales in March and April). We investigate which covariates (species, length, reproductive class or sex, day of the year and depth interval) are linked to the intensity of recent bites, and discuss what the implications of the scarring patterns are for our knowledge of the distribution and migrations of the whale species involved.

Throughout this paper we have used the terms “predation” and “prey” in regard to the relationship between the biting agent and large whales, rather than “parasitism” and “host”. We do this because there is no evidence of the relationship between biting agent and victim being anything other than transitory, even though the interaction is highly unlikely to result in the death of the victim.

The available evidence, presented in S1 Text, including wound characteristics and anatomy of the candidate biting agents, would indicate that the most likely perpetrator of bites on large whales is an oceanic shark belonging to the genus *Isistius*, possibly *Isistius brasiliensis*, that has a circumpolar distribution in offshore tropical waters. The incidence of fresh bites and the discovery of a reverse scoop in a sperm whale stomach at the Donkergat whaling station (S1 Text and S1 Fig) would certainly suggest that attacks must occur within the vicinity of the whaling ground. Based on this evidence, we conclude that a cookie-cutter shark of the genus *Isistius* is the agent responsible for most of the crater-like wounds seen on large whales in South African waters.

## Materials and Methods

### Data collection

**Fig 2:**
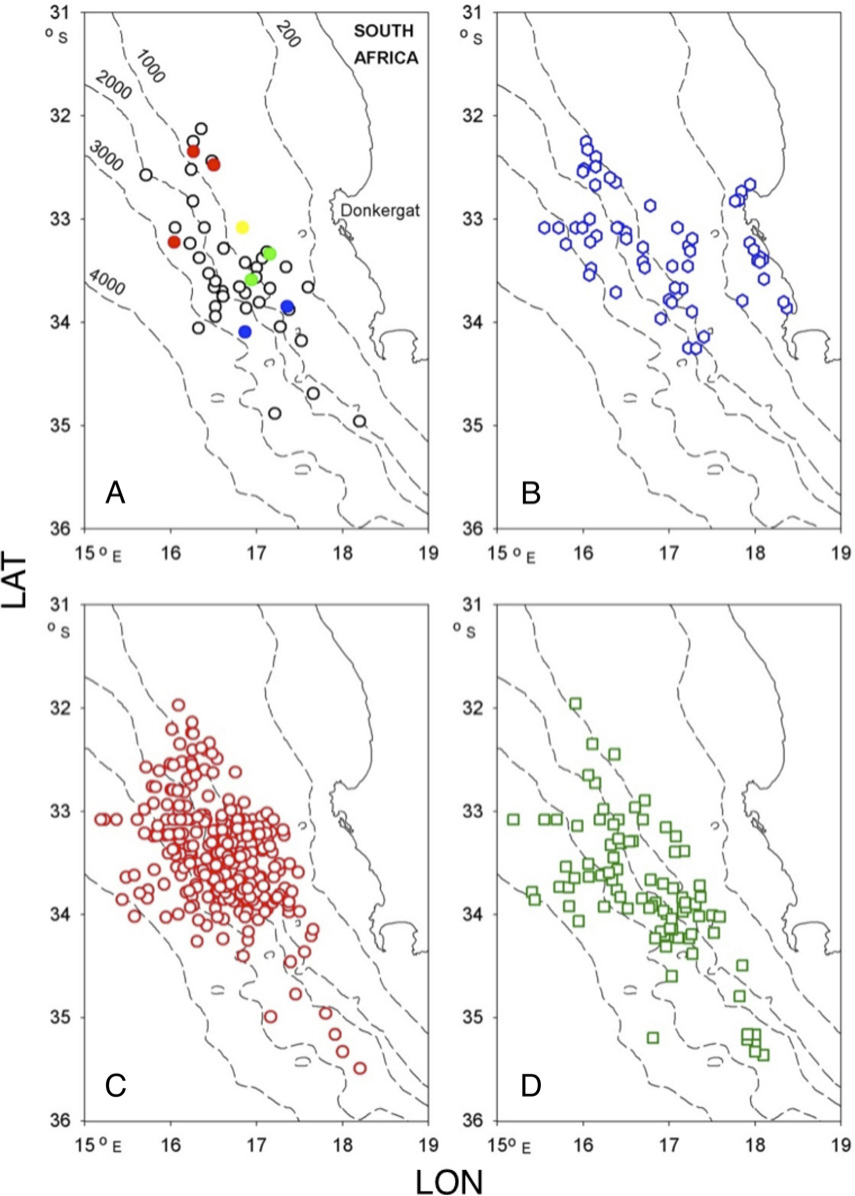
Whaling ground from Donkergat, South Africa, showing 1963 catch positions of A fin (black), blue (blue), humpback (red), minke (yellow) and killer whales (green); B Bryde’s whales; C sei whales and D sperm whales in relation to bathymetry (in m).

Between 2 March and 31 October 1963, a total of 1,737 whales were examined at the Donkergat whaling station in Saldanha Bay, South Africa (Fig 2, Table 1). As part of this examination, individual whales were scored for the presence of bitemarks and scars now believed to be attributable to a cookie-cutter shark of the genus *Isistius*. No permits were required for collection of the data by PBB, who was a research officer with the Fisheries Development Corporation of South Africa at that time. However, permission to examine carcasses, while they were on the platform prior to flensing, was granted by the commercial use company (Saldanha Whaling Co. Ltd).

**Table 1:** Whales examined at the Donkergat whaling station, South Africa, 1963

Species	No. landed	No. examined for *Isistius* scarring	No. examined for recent bitemarks	No. examined for healed scars
Sperm	852	850 (620)^a^	846(618)	0
Killer	2	2		2
Humpback	3	2	1	2
Blue	2	2	1	2
Fin	56	55	55	55
Sei	728	718	688	693
Bryde’s	93	92	80	88
Minke	1	1		1
Total	1,737	1,722 (1,492)	1,671 (1,443)	843

^a^ Values in brackets refer to alternative assumption regarding “missing” records for sperm whales (S2 Text).

**Fig 3:**
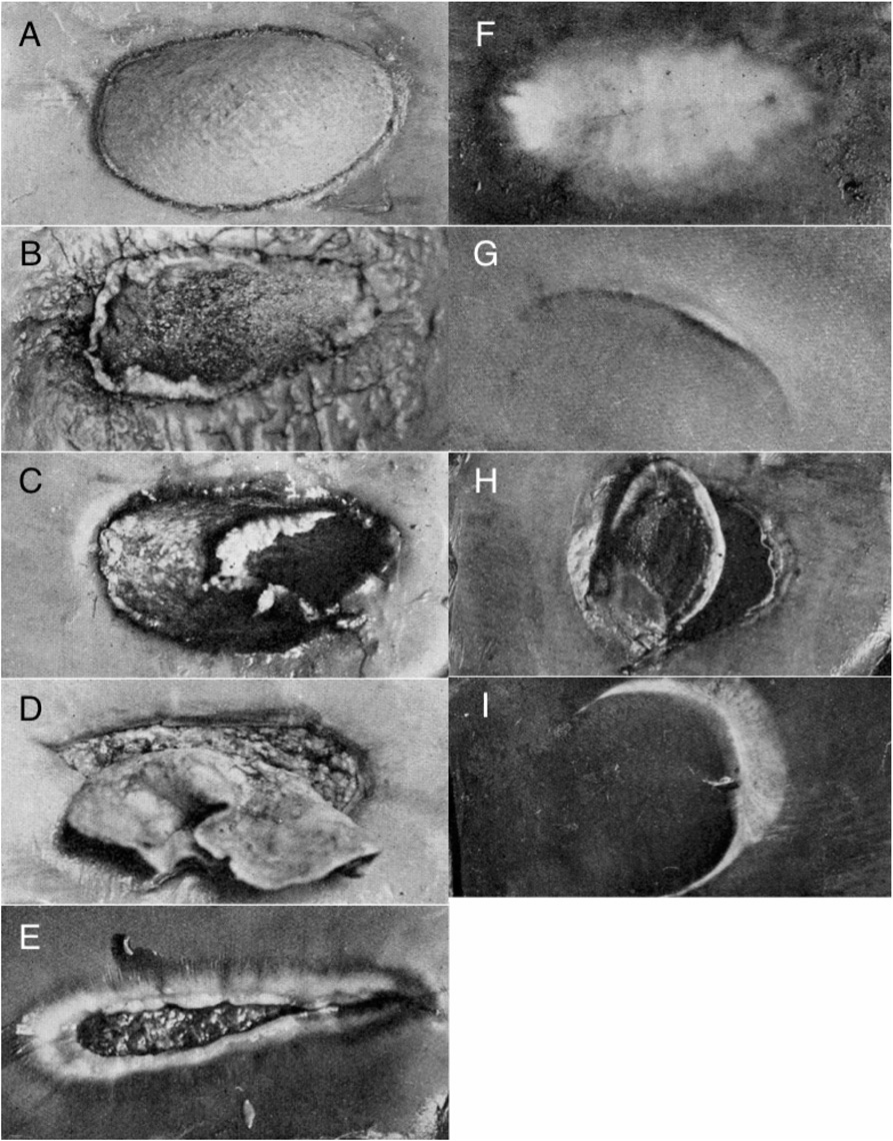
Categorisation of bite wounds on large whales at Donkergat whaling station (from [6]): A pink/fresh oval, B—E healing ovals, F healed oval, G attempted half-scoop, H open half-scoop and I healed half-scoop.

Unhealed or open bitemarks were counted and classified as “recent” (edges of the wound well-defined and the exposed blubber either still pink or with no signs of healing: Fig 3A) or “healing”/“brown” (edges of the wound poorly defined and the exposed tissue granulating: Fig 3B–3E): “half-scoops” or incomplete bites were recorded separately (Fig 3G–3H). In sperm whales, where unhealed bites were relatively few, the location of each bitemark was recorded as in 1 of 12 areas: front of head, side of head, top of head, neck/shoulder, chest, flipper, flank/side of body, belly, umbilical region, genital region, peduncle, tail. Such detail was rarely possible for other species because of the numbers of bites involved.

Healed bitemarks or scars (Fig 3F–3I) were subjectively classified as few, moderate or many: latterly intermediate categories were introduced, such as few-moderate. Such categorization was only possible for baleen whales, as healed bitemarks in sperm whales were apparently re-pigmented so rapidly that they became essentially invisible.

Because of the posture of the whale as it lay on the platform prior to flensing, approximately 30% of its body was in contact or near-contact with the deck at any one time and thus inaccessible for inspection, and only revealed once the whale was turned over after the blubber had been removed from the other side. This meant that two inspections of each whale were needed to obtain a full count of unhealed bitemarks. Sometimes only one such inspection was possible, and because of the asymmetry in size of the 2 “sides” examined, any counts of unhealed bitemarks made from one side only have been discounted in analysis of bitemark incidence. Data from one side only however have been used in assessing the relative proportion of fresh/recent to other unhealed bitemarks and in the categorization of healed scarring.

Body lengths of all whales landed were measured in feet and inches to the nearest foot, as dictated by [19], and these lengths have been retained here without conversion to their metric equivalents to avoid the impression of spurious accuracy.

Catch locations were originally recorded as bearings and distances from the whaling station, but these have been converted here to latitude and longitude. Given the inaccuracies of contemporary navigation, we have considered it sufficient to assign each catch location to a depth interval rather than a specific depth. We used bathymetric data from [20] to allocate locations to 6 depth intervals, 0–200 m, 200–1,000 m, 1,000–2,000 m, 2,000–3,000 m, 3,000–4,000 m, and 4,000–5,000 m: in practice there were so few captures over 4,000 m that for analysis they were combined with those between 3,000 and 4,000 m (Fig 3).

Day of the year was calculated from 1 January as day 1.

Bryde’s whales caught off Donkergat are believed to belong to 2 different populations, one occurring inshore and the other offshore [21,22]. For the purposes of this analysis, all whales taken in <200 m water depth were assumed to be “inshore” and all those in deeper water to be “offshore”.

For baleen whales, male whales were classified from testis samples as either immature or mature based on histological criteria. Females were classified as either immature or mature based on whether they had ovulated (at least 1 corpus luteum or albicans in the ovaries), while mature females were classified as pregnant (primigravid or multigravid), lactating or resting, using the presence of a foetus, milk in the mammary gland, or neither respectively: ovulating females were included in the resting category. Sperm whales were classified into 4 social groupings, as small males (<40 ft long), medium males (40–45 ft long), large males (>45 ft long) and females, based on their social organization [23].

### Statistical analysis

The bitemark count data were analysed separately for each species of whale due to the presence of different reproductive classes and apparent relationships between variables in the raw data. In all cases Generalized Additive Models (GAMs) were fitted to the count data for the total number of unhealed bitemarks using the mgcv library in R [24]. This count represents intensity of predation. The proportions of total unhealed bitemarks that were fresh/recent were also analysed for sei and fin whales (inadequate data were available for sperm and Bryde’s whales). The proportion of fresh bites was used to assess trends in the seasonality of predation. Model selection, based on all possible subsets of covariates, was carried out using the dredge function in the MuMIn library [25] based on Akaike weights [26] (using the Akaike Information Criterion for finite sample sizes for quasi-likelihood models, QAICc), diagnostic plots and the percentage deviance explained. Differences in QAICc can be interpreted as the relative weight of evidence or support for each model.

#### Total number of unhealed bitemarks

The count data from all species were overdispersed (higher variability in the data than expected) so models with a quasipoisson error structure and a log link function were fitted (S1 Code).

We present the candidate models for each whale species in Table 2. For Bryde’s whales a model was only fitted to the bitemark counts from specimens of the offshore type, because none of the 19 whales harvested in water shallower than 200m showed any bitemarks. For sperm whales, models were fitted both for Option A and Option B regarding the interpretation of missing data (S2 Text).

**Table 2:** Candidate covariates for regression models of the total number of unhealed bitemarks, and the proportion of unhealed bitemarks that were recent on four whale species.

Species	Candidate covariates				
	Day ^a^ (integer)	Reproductive class (categorical)	Sex (binary)	Depth interval (ordinal ^b^)	Length (ft) (integer)
Sei	61–303	female, male (small / medium / large)	male, female	2–6	26–53
Fin	134–304	male (immature / mature), female (immature / mature)	male, female	2–5	39–72
Offshore Bryde’s	85–301	male (immature / maturing / mature), female (immature / resting / lactating / mature)	male, female	2–5	32–51
Sperm ^c^	61–303	female, male (small / medium / large)	male, female	2–6	26–53

^a^ Julian day.

^b^ 1: 0–200 m; 2: 200–1,000 m; 3: 1,000–2,000 m; 4: 2,000–3,000 m; 5: 3,000–4,000 m; 6: 4,000–5,000 m.

^c^ In sperm whales social grouping was considered a more appropriate grouping than reproductive class. In this species, body length was confounded with social grouping so only the latter was included, as a factor variable. Sex was not fitted as a separate covariate as it was included in the variable for social grouping.

#### Proportion of unhealed bitemarks that were recent

The data on the proportion of total unhealed bitemarks that was recent on each whale were underdispersed (less variability in the data than expected), and included 0 and 1, so models were fitted with a quasibinomial error structure and a logit link function (S2 Code). Only the data from fin and sei whales could be used for this analysis. The rapid repigmentation of recent bites on sperm whales, and the lack of signal in the dataset for offshore Bryde’s whales rendered this analysis uninformative in sperm and Bryde’s whales.

Candidate models for the proportion of unhealed bitemarks that were recent in sei and fin whales included the same covariates as the models for the total number of unhealed bitemarks (Table 2).

## Results

### Observations of the total number of crater wounds on large whales

**Fig 4:**
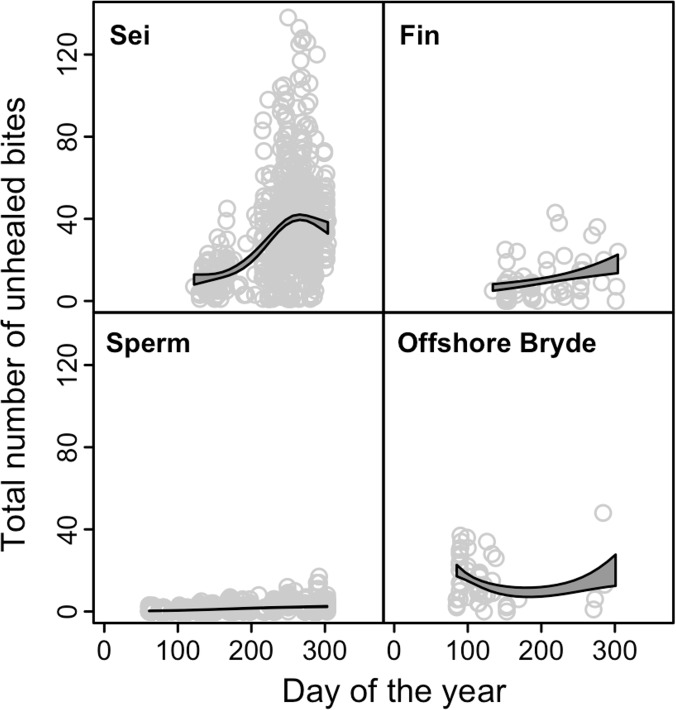
Number of unhealed bites on individuals of four whale species landed at Donkergat, 1963, plotted against date of capture.

Plots of the numbers of open wounds as a function of days since the beginning of the year revealed a positive relationship between numbers of open wounds and days since the beginning of the year in sei, fin and sperm whales (Fig 4). For offshore Bryde’s whales the number of wounds was already high when the season opened (in March), with little sign of any positive relationship with date thereafter.

Sei whales were recorded as carrying 0–138 unhealed bitemarks ($\bar{x}$ ± SE = 33.89 ± 0.96, *n* = 671). Only 32 or 0.1% were half-scoops: 10 of these were distributed on the tail and 1 each on the peduncle, dorsal fin, mandible, side of head and rostrum (the location of the others was not recorded).

We present the four models with highest support for each species in Table 3.

**Table 3:** Models for the total number of unhealed bites on all whale species, including differences in the QAICc scores (ΔQAICc) and Akaike weights.

Species		Covariates	ΔQAICc		Weight	
	Model 1	Reproductive class + Depth interval + Day	0.00		0.546	
Sei	Model 2	Reproductive class + Depth interval + Length + Day	2.06		0.195	
	Model 3	Reproductive class + Day	2.10		0.191	
	Model 4	Reproductive class + Length	4.18		0.068	
	Model 1	Depth interval + Length	0.00		0.298	
Fin	Model 2	Depth interval + Length + Day	0.64		0.216	
	Model 3	Length + Day	2.27		0.096	
	Model 4	Depth + Length + Sex	2.49		0.086	
	Model 1	Day + Length + Sex	0.00		0.460	
Offshore	Model 2	Day + Length	1.50		0.217	
Bryde’s	Model 3	Day + Length + Depth interval	2.86		0.110	
	Model 4	Day + Length + Sex + Depth interval	3.30		0.088	
		Option ^a^	A	B	A	B
	Model 1	Day + Social grouping + Depth interval	0.00	0.00	0.574	0.855
Sperm	Model 2	Day + Social grouping	1.05	3.89	0.339	0.122
	Model 3	Day + Depth interval	4.50	7.44	0.061	0.021
	Model 4	Day	6.14	12.71	0.027	0.001

^a^ Option A assumes that missing data imply no recent scars. Option B assumes that missing data imply no observations were made (S2 Text).

**Fig 5:**
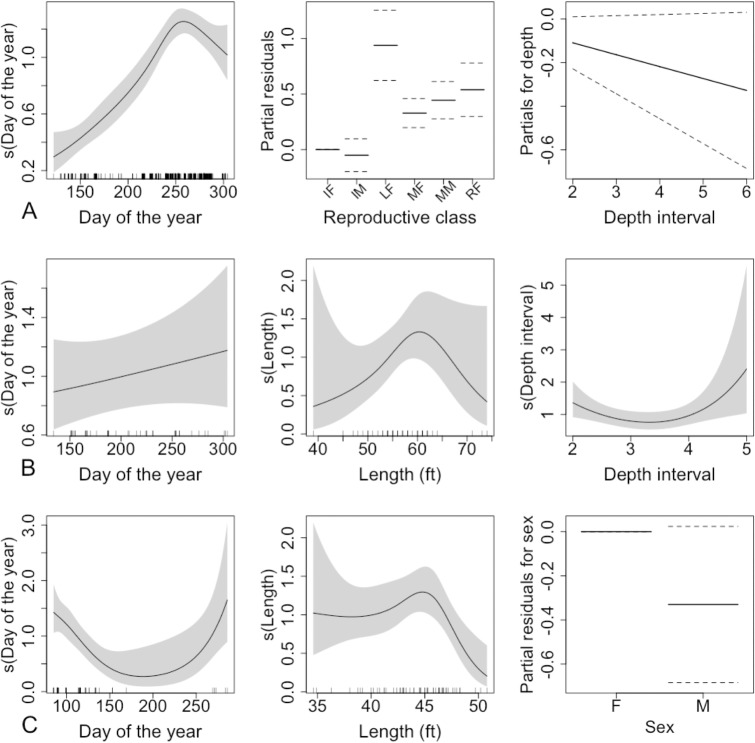
The relationship between total unhealed bitemark counts and each explanatory variable in the model for baleen whales. For sei whales (A) these include a smooth term for day of the year, a factor variable for reproductive class plotted against the partial residuals for that variable, and a linear term for depth interval; for fin whales (B) a linear term for day of the year and smooth terms for body length and depth interval; and for Bryde’s whales (C) smooth terms for day of the year and body length, and a factor variable for sex. The grey shading in plots of smooth functions, and the dotted lines in the other plots extend two standard errors either side of the fitted relationship.

There was support for 4 of 16 possible models for sei whales. The model with highest support (Table 3) explained 28% of the variability in the data (deviance) (Fig 5A). Lactating females had the highest number of bitemarks, followed by resting females, and mature adults of both sexes. Immature animals had the lowest number of bitemarks. The counts peaked during the last quarter of the year and there was a weak, decreasing relationship with depth.

Restricting the analysis to the months of September/October made it possible to reduce the effect of day of the year and focus on the difference between reproductive classes in sei whales. A Tukey HSD Test (following a significant One Way ANOVA, *F*_4,221_ = 5.89, *P* < 0.01) showed that lactating sei whale females had significantly more recent bites than any other class other than resting females, while resting females had significantly more bites than multigravid females (S1 Table).

Fin whales were recorded as carrying 0–43 unhealed bitemarks ($\bar{x}$ ± SE = 10.3 ± 1.4, *n* = 55). Only 2 or 0.4% were half scoops, 1 of which was on the mandible.

Models for fin whales with all possible combinations of covariates received some support (15 out of 16 possible models). The top two models (Table 3) had practically identical QAICc scores although there was a difference in the degree of support (30% vs 22%). These two models explained 30% and 32% of the variability in the data (deviance explained), respectively (Table 3). The results based on the model explaining the highest percentage of deviance are presented (Fig 5B). All fitted relationships were diffuse in this species, however there was a linear, increasing trend with day of the year, a peak in bites at intermediate body length, and the most bitemarks occurred at the shallowest and deepest depths.

For Bryde’s whales unhealed bitemarks were only recorded on 2 of 22 whales taken inshore, being 1 and 2 in number respectively i.e. an average of 0.1 per whale: no half scoops were recorded. The remaining Bryde’s whales that were taken offshore were recorded as carrying 0–46 unhealed bitemarks ($\bar{x}$ ± SE = 15.2 ± 1.5, *n* = 58). Only 1 half scoop (or 0.1% of the total) was recorded, on the tail. Only the counts on whales taken offshore were modelled.

Due to small sample size for this species, sex was used instead of reproductive class. There was some support for models with all possible combinations of the covariates. The best model explained 33% of the variability in the data (deviance explained) (Fig 5C and Table 3). In this species, bitemark counts were highest early and late in the year, and lowest in the second and third quarters. There was a diffuse relationship with body length, whereby bitemarks peaked towards the higher end of the range. There was weak evidence of a sex effect, which suggested lower mean counts on males.

Sperm whales were recorded as carrying 0–17 unhealed bitemarks ($\bar{x}$ ± SE = 1.3 ± 0.1, *n* = 846, Option A, or 1.8 ± 0.1, *n* = 618, Option B) (S2 Text and S3 Text). Of these, 38 (or 3.3%) were half-scoops: 3 of these were on the front of the head, 1 on the side of the head, 1 on the top of the head, 2 on the head (unspecified), 2 on the chest, 2 on the back, 2 on the flanks, 1 near the genital aperture, 1 on the peduncle and 1 on the tail (the location of the others was not recorded).

**Fig 6:**
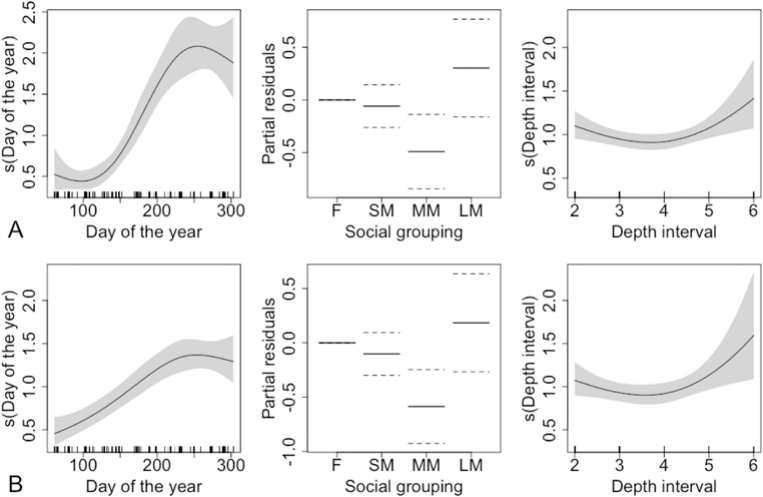
The relationship between total unhealed bitemark counts in sperm whales and each explanatory variable in the model, including smooth terms for day of the year and depth interval, and a factor variable for social grouping. In the middle column, the y-axis refers to the partial residuals for social grouping. The grey shading in plots of smooth functions, and the dotted lines in the other plot extend two standard errors either side of the fitted relationship. The upper figure (A) follows Option A and the lower figure (B) Option B in the interpretation of missing counts (S2 Text).

Models were fitted for both options (A and B). The models with highest support explained 20% of the variability in the data for Option A and 13% for Option B (Fig 6).

In the models for both options, bitemarks on spern whales peaked in the last quarter of the year. There was a stronger depth effect under Option B than Option A, though the pattern was the same with moderately lower numbers of bitemarks at intermediate depths. The pattern of bitemarks with respect to social grouping and sex was the same under both options, and did not follow body length. Large males had the largest mean fitted number of bitemarks while medium-sized males had the lowest. There was an increasing trend in the size of the residuals with the fitted values in all models, which suggests that the model does not adequately explain the pattern in the data for this species. This could be due to missing covariates, or misspecification of the model or error structure. Fitting a model with a Negative Binomial distribution for the errors did not improve the fit. In addition, the range of the fitted values was much smaller than the range of the observed data, which is common in models with a log link.

Large males exhibited more unhealed bites than medium-sized males (Table 4), with no other social groups exhibiting significant differences.

**Table 4:** Average number of unhealed bites on sperm whales of different size and sex classes, Donkergat, South Africa, 1963.

Sex and size class	*n*		Mean number ± SE		Tukey HSD Test	
	Option ^a^					
	A	B	A	B	A	B
Females (F)	292	206	1.42 ± 0.12	2.01 ± 0.15		
Small males (SM)	410	313	1.46 ± 0.10	1.92 ± 0.12		
Medium males (MM)	118	81	0.72 ± 0.12	1.05 ± 0.17	<LM*	<LM**
Large males (LM)	26	18	1.65 ± 0.56	2.39 ± 0.75		

*P<0.05.

** *P*<0.01.

^a^ Option A assumes that missing data imply no recent scars. Option B assumes that missing data imply no observations were made (S3 Text).

### Number and proportion of unhealed bites that were recent

The number of unhealed wounds that were classified as recent bites ranged from 0 to 11 on sei whales ($\bar{x}$ ± SE = 1.04 ± 0.06, *n* = 532), from 0 to 5 on fin whales ($\bar{x}$ ± SE = 1.33 ± 0.21, *n* = 39), and from 0 to 6 on offshore Bryde’s whales ($\bar{x}$ ± SE = 1.23 ± 0.21, *n* = 35). The incidence of recent bites was significantly different between the three species with Bryde’s whales being more likely to receive >2 bites, and less likely to receive no bites than other species (Chi-square test, χ^2^ = 11.16, *P* = 0.0248).

The number of unhealed wounds that were classified as recent bites on sperm whales ranged from 0 to 3 ($\bar{x}$ ± SE = 1.18 ± 0.08, *n* = 60). However the very low proportion of individuals (8.3%) for which data were recorded (see Material and Methods), raises doubts over whether negative records were adequately captured for this species.

We present the four models with highest support for each species in Table 5.

**Table 5:** Candidate models for the proportion of unhealed bites that were recent in all whale species.

Species		Covariates	ΔQAICc	Weight
	Model 1	Day + Reproductive class + Depth interval	0.00	0.572
Sei	Model 2	Day + Reproductive class + Depth interval + Length	0.84	0.376
	Model 3	Day + Length + Depth interval	5.91	0.030
	Model 4	Day + Length + Sex + Depth interval	7.44	0.014
	Model 1	Day	0.00	0.215
Fin	Model 2	Day + Depth interval	0.00	0.215
	Model 3	Day + Length	0.42	0.174
	Model 4	Day + Depth interval + Length	0.68	0.153

**Fig 7:**
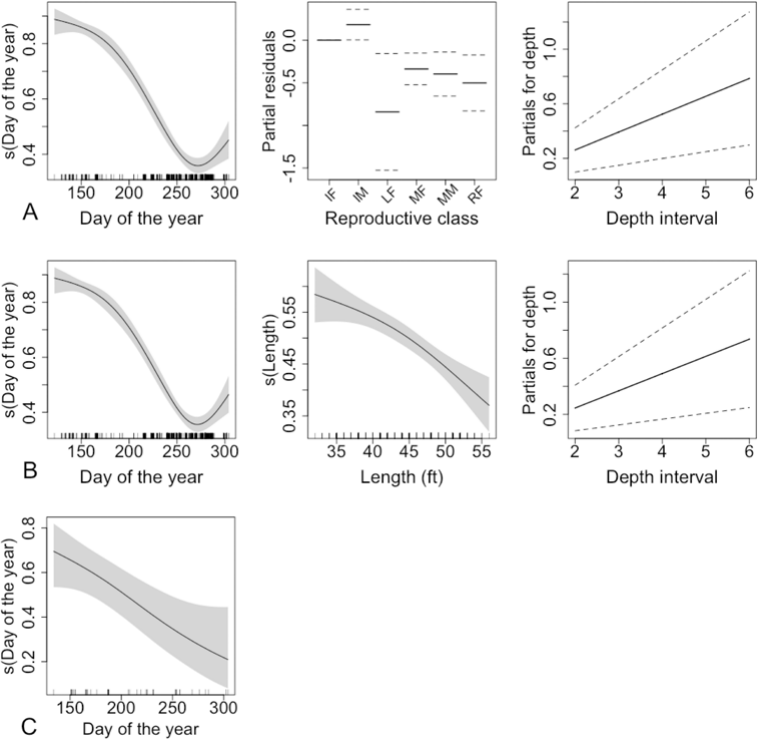
The relationship between proportion of unhealed bitemark counts that are recent in sei (A, B) and fin (C) whales and each explanatory variable in the model, including smooth terms for calendar date and depth interval, and a factor variable for reproductive class. In the middle column, the y-axis refers to the partial residuals for reproductive class. The grey shading in plots of smooth functions, and the dotted lines in the other plot extend two standard errors either side of the fitted relationship.

While modelling the proportion of unhealed bites that was still fresh in sei whales, we found some support for 6 out of 16 possible models. Length and reproductive class are confounded explanatory variables, since mature animals will be bigger, so it is inappropriate to include both in the same model. We present models including each term for comparison (Fig 7A and 7B, Table 5). Both models explained 39% of the variability in the data (deviance explained).

According to the model with the highest support, the proportion of recent bitemarks decreased over the course of the year, with a slight upturn in proportion in the last quarter, and there was a monotonic, increasing relationship with depth interval. In the model including length, there was a monotonic, decreasing relationship between the proportion of recent bitemarks and this explanatory variable. This is consistent with what we see in the model including reproductive class instead of length, where mature adults of both sexes had significantly lower proportions of recent bitemarks compared to the reference class (immature females) and there was little difference between immature animals of the two sexes. We note that lactating females had by far the lowest proportion of recent bitemarks in this species, significantly lower than immature females, which were the baseline reproductive class in the model.

We found some support for 14 out of 16 possible models for fin whales. The relationship between the response and depth was found to be a flat line while excluding it made no difference to the relationship with day of the year, therefore that model was not considered. The trend in bitemarks over the course of the year, based on the model with day of the year as the sole explanatory variable (Table 5), the proportion of recent bitemarks decreased over the course of the year (Fig 7C). Both models (with and without depth) explained 17% of the variability in the data (deviance explained).

The location of 253 recent bitemarks was recorded in 13 regions of the body for 169 sperm whales landed at Donkergat (S2 Table). The side of the head was the region receiving the highest proportion of bitemarks (21.3%), while the head altogether received 36.7% of all bites whose position was recorded. Of the 54 bites recorded on the side of the head, 12 (or 22%) were located at the corner of the mouth or on an upper lip. Nearly half (48.6%) of the bites were in the anterior half of the body (i.e. from the shoulder/flipper area forwards).

Incomplete (or C-shaped) bites were also differently distributed over the body in baleen and sperm whales (S3 Table). In baleen whales, 70.6% of such bites were located on the tail or peduncle, significantly more so than in sperm whales (12.5%; Chi-square test, χ^2^ = 9.13 (Yates correction), *P* < 0.01), where they were more evenly distributed over the body.

#### Distribution of whales with recent bites in relation to bathymetry

To investigate whether the biting agent was restricted to a particular water depth, the frequencies of whales recorded as with or without recent bites have been compared at each depth interval (S4 Table): whales where presence or absence of recent bites was not recorded have been omitted. The data indicate a complete lack of any recent bites on whales taken in water shallower than 200 m, but a sharply increasing incidence of such bites in whales taken in deeper water, reaching 83% in water depths exceeding 3,000 m. In addition, the numbers of recent bites on each whale bitten were significantly different between depth intervals over 200 m (One way ANOVA, *F*_3,391_ = 4.38, *P* < 0.01), with a Tukey HSD test indicating that whales taken in waters over 3,000 m in depth had a significantly higher number of bites per whale than those taken in shallower water (S4 Table).

## Discussion

Evidence presented above would indicate that the most likely perpetrator of the bites on large whales is an oceanic shark belonging to the genus *Isistius*, possibly *Isistius brasiliensis*, that has a circumpolar distribution in offshore tropical waters. The southern limit to its distribution in the South Atlantic is poorly known but has been described as 34–35°S [14] while specimen records (some poorly documented) extend to about 37°S. The incidence of fresh bites and the discovery of a reverse scoop in a sperm whale stomach at the Donkergat whaling station would certainly suggest that attacks must occur within the vicinity of the whaling ground.

Although a small number of covariates were used, in addition to the fact that they are acting as proxies for the drivers of the distribution of animals in space, the model results illuminate certain trends in the total and recent unhealed bitemark data. Day of the year was an important explanatory variable in models of total unhealed bitemark counts for all species, and depth alike for all species except offshore Bryde’s whales. Length was retained in the most likely models for fin and Bryde’s whales, but was replaced by reproductive class in sei and social grouping in sperm whales. The importance of day of the year reflects the obvious tendency in most species for open bitemarks to increase as the season progressed, while the influence of body length in the two largest baleen species suggests that in some species, target size may be a factor in attracting predation. The relationship between total unhealed bitemarks and sex, reproductive class or social grouping in these models likely reflects the respective geographic distribution and ranging patterns of within these whale species.

Systematic data on the location of recent bites on the body were not collected for baleen whales. The distribution of healed scars in North Atlantic sei whales has been described as “most frequent on the sides of the body somewhat below the middle, and occur also on the tail, but may be found, when they occur to any great extent, distributed singly right up to the center of the back, and extending as far as the end of the lower jaw.” [27]. On southern blue and fin whales they were described as mainly on the posterior end of the body, and at Saldanha Bay unhealed bitemarks were recorded on the flanks and tail [6]. According to [28], the scars occur most frequently (in blue and sei whales) on the caudal peduncle and sides of the body, with somewhat more on the lower than upper half of the body. A figure of offshore Bryde’s whales landed at Donkergat clearly shows how targeted the peduncle area can be in baleen whales [13].

The incidence of open bites on baleen whales at Donkergat from autumn onwards provides a potential tool for investigating patterns of migration for these species, as do assessments of the degree of accumulation of healed scars. In these respects, the qualitative differences between the 2 populations of Bryde’s whales and between them and the other 2 baleen whale species, sei and fin whales, broadly reflect what is currently known about their distribution and movements. Models for the proportion of total unhealed bitemarks that were recent in sei and fin whales both included day of the year and showed an inverse form, particularly clear in the case of sei whales, compared with the total number of unhealed bitemarks. Almost all open bitemarks in sei whales were fresh early in the season (ca. day 145–165), consistent with the recent arrival of the species on the whaling grounds from higher latitudes. For fin whales, there was a similar decreasing trend between the proportions of unhealed bitemarks that were recent and day of the year, but the pattern was less clear, possibly due to the smaller sample size for this species. The small increase in the proportion of open wounds that were fresh at around day 300 in sei whales is difficult to explain but may result from variability in the southern migration route, with some individuals passing through a high *Isistius* concentration zone (perhaps more offshore–see trend with depth) in the vicinity of the whaling ground towards the end of the southern migration.

The low numbers of healed scars and scarcity of unhealed bites (with a zero proportion recent) on inshore Bryde’s whales reflect their habitat (virtually exclusively in water <200 m deep, outside of the range of *Isistius*) and lack of major seasonal movement [29]. By contrast, offshore Bryde’s whales exhibited a large number of healed scars, lack of seasonal trend in the numbers of unhealed bites, and relatively high number of these that are recent. This presumably reflects both the offshore habitat and abbreviated latitudinal range of migration exhibited by offshore Bryde’s whales, such that they probably live year-round within the habitat of *Isistius*, migrating from equatorial regions (principally between Gabon and northern Angola) in winter to more southerly latitudes (off Namibia and South Africa) in summer [30,31].

In sei and fin whales the degree of accumulation of healed scars is intermediate between inshore and offshore Bryde’s whales, consistent with the belief that these are largely offshore species but that they migrate seasonally to the Southern Ocean where they would receive relief from *Isistius* attacks. The difference in the degree of scarring between fin and sei whales may partly arise from differences in the age composition of the catch: only 9.1% of the fin whale catch was sexually mature compared to 45.6% of the sei whales taken. If scars accumulate over many years, then one would expect older animals to show more scars than younger ones. Both species show an increasing number of open wounds from late autumn onwards, consistent with the concept that they have spent the preceding summer months in an *Isistius*-free region to the south and are being regularly bitten while in warmer waters. The higher than expected frequency of zero recent bites and lower than expected frequency of 2 such bites compared to offshore Bryde’s whales, suggest a lower biting rate of sei and fin whales. This is unlikely to be the result of selection by the biting agent, given that sei and Bryde’s whales at least are very similar in size and appearance. Consequently, it is more likely to be due to temporal or spatial distribution differences and perhaps diving behavior that lead to differences in exposure risk, though differences in diving behavior between the baleen species considered here and within the study area are not well known.

In sei whales, the importance of reproductive class in the model for total unhealed bites presumably represents the greater accumulation of unhealed bites seen on lactating females: this may arise from a higher surfacing or respiratory rate for cow-calf pairs [32–34], meaning more time spent at or near the surface where we believe attacks may take place. Alternatively, it could mean a longer residence time for near-term/lactating females in warmer waters, although there is no empirical evidence to support this. Such an effect was presumably not detectable in fin or Bryde’s whales because of the smaller sample sizes. In sperm whales, the significance of social grouping seems to arise from the higher biting rate on large versus medium-sized males: we speculate that this may arise from a longer residence time in lower latitudes for socially mature individuals associating with schools of females [23]. If so, this is a clue to one of the biggest gaps in our understanding of sperm whale behavior, the movements of mature and maturing males [35].

The biting agent (if it is indeed one and the same species) seems to interact differently with sperm and baleen whales. Firstly, the average number of recent bites is about an order of magnitude less than for any of the baleen whale species (other than the inshore Bryde’s population). Target areas also differ, with bites being concentrated on the rear half of the body in baleen whales but occurring more diffusely over the body in sperm whales, with some concentration on the head region. Furthermore, unsuccessful attacks (resulting in an incomplete bite) seemingly occur more often on sperm whales, and on any region of the body rather than being concentrated on the tail and peduncle region. The reasons for these differences are unknown. The authors of [36] proposed that the distribution of bitemarks over the body of (mainly) small odontocetes (concentrated on the sides (40%), belly (20%) and head (20%), with none on the peduncle) can be attributed to the more mobile nature of the rear half of the body, but this seems unlikely to account for the different distribution patterns between sperm and baleen whales. Fewer open wounds may mean that sperm whales are a less favored or less accessible target, or that wound-healing may be very much faster than in baleen whales. The different distribution of attacks over the body could simply reflect the fact that the sperm whale’s head is relatively more massive than that of a baleen whale, but it could also reflect the unusually corrugated nature of the surface of the skin over much of the rest of the sperm whale’s body. These corrugations are present on the back, flanks from the flippers to the tail flukes, and to a lesser extent on the ventral surface from the lower chest to the umbilicus [11]. The smooth skin on the head (and especially the spermaceti organ) might represent a more appropriate target for a small biting shark such as *Isistius*. The greater and more diffuse occurrence of incomplete bites could also be a consequence of the structure of sperm whale blubber, in which the dermis is especially thick and the ligature of the collagen fibres is dense [37], presumably making incisions into it more difficult. Characteristics of dermal tissue have been suggested to influence *Isistius* biting rates in some species of fish [38].

The clustering of bites near the angle of gape or on the upper lip of sperm whales would seem to represent a hazardous target for *Isistius*, given that sperm whales in the region are at least partially piscivorous [39]. The authors of [40] speculate that the white teeth and buccal lining of the sperm whale’s mouth, combined with bioluminescence stimulated by jaw movements, could act as a lure for organisms attracted to light. This in turn could directly or indirectly attract *Isistius* to the proximity of the mouth sometimes ending up as prey perhaps while trying to attack. This possibility if also mentioned by [41] while [42] document a specimen of *Isistius* found in the stomach of a tuna. The finding of a scoop but no biting agent in a sperm whale’s stomach could mean that the victim reacted to such an attack, causing the shark to regurgitate its spoils. An alternative scenario would be that the sharks themselves are acting as lure [43] for sperm whales and other predators which are targeted by *Isistius*, which are then sometimes taken. Another possibility still, is that the sharks themselves were the target of predation by the sperm whale. [44] refers to the remains of two small bottom-dwelling squaloid sharks *Scymnodon* (*obscurus* or *squamulosus*) from the stomach of a sperm whale taken at the Durban whaling station in August 1969, while [45] refer to four semi-digested *Scymnodon obscurus* taken by a sperm whale in the same locality in August 1971: the latter sharks ranged from 550 to 720mm in upper caudal length, or of a similar size to *Isistius*. The distribution of bites on the head of sperm whales could, in that case, reflect a response to predation attempts.

## Conclusions

We present compelling evidence that *Isistius* sp cookie-cutter sharks are the causative agents of crater wounds on large whales, based on data collected from whales landed at Donkergat over 8 months in 1963. Our analysis of the counts of unhealed bites and assessment of the degree of healed scarring on large whales presented in this paper represent the first attempt to document the degree of predation by the biting agent in lower latitudes, and to examine how this predation varies between species, size/reproductive categories and over time and a proxy for space. Further interpretation of the biting patterns, including modeling of the biting rate and healing process, will be the subject of future work. Our results support what is known about the movement of sei, fin and offshore Bryde’s whales. Furthermore, they offer new insights into one of the least known areas of sperm whale behavior, the ranging patterns of mature and maturing sperm whales, namely that medium sized (maturing) males appear to spend less time at lower latitudes and within the range of *Isistius* than large (mature) males.

## Supporting Information

S1 Code Code for fitting GAMs to total number of unhealed bitemarks. (DOCX)

S1 Dataset Minimal dataset. (RDATA)

S1 Fig Scoop of blubber found in the stomach of a sperm whale at Donkergat whaling station, 2 September 1963 (platform # 1066), showing: A—dorsal, B—lateral, and C—ventral views (scale in cm). (TIF)

S1 Table Average numbers of unhealed bites on 226 mature sei whales of different reproductive classes examined at the Donkergat whaling station, South Africa, September/October 1963, with results of Tukey HSD Test. (DOCX)

S1 Text A review of the identity of the biting agent: evidence for and against Isistius as the biting agent. Including a comparison of wounds made by *I*. *brasiliensis* and *I*. *plutodus*, the biting technique of Isistius and a case study of a “reverse scoop” found in a sperm whale stomach and a review of other potential biting agents.
(DOCX)

S2 Code Code for fitting GAMs to the proportion of unhealed bitemarks that were recent. (DOCX)

S2 Table Location of unhealed bitemarks on 169 sperm whales examined at the Donkergat whaling station, South Africa, 1963. (DOCX)

S2 Text Interpretation of missing records. (DOCX)

S3 Table Location of unhealed incomplete bites on whales examined at the Donkergat whaling station, South Africa, 1963. (DOCX)

S3 Text Account of an outlying observation of a sperm whale with a large number of unhealed bitemarks. (DOCX)

S4 Table Incidence of recent bites on large whales landed at the Donkergat whaling station, South Africa, 1963, and mean number of bites per whale bitten, by depth interval. (DOCX)
