# Supplementary material for: Identifying the “demon whale-biter”: Patterns of scarring on large whales attributed to a cookie-cutter shark Isistius sp
Source: PLoS One. 2016 Apr 7;11(4):e0152643. doi: 10.1371/journal.pone.0152643 (PMC4824425; doi:10.1371/journal.pone.0152643)
Supplement: S1 Code — (DOCX) [file pone.0152643.s001.docx]

S1 Code. Code for fitting GAMs to total number of unhealed bitemarks

Models for total counts of unhealed bitemarks were fitted with a quasipoisson error structure and a log link function. Where non-linear relationships between the response and explanatory variables were present, these terms were fitted as smooth functions using the “cs” basis function with shrinkage [1]. This allows the effect of covariates to be shrunk to zero if there is no relationship with the response. Below is the code used to total unhealed bites each whale species and the code used to produce the plots of the fitted relationships for the models with highest support. The number of knots was checked following routines documented in the mgcv library [2].

# Sei whales

## Model

library(mgcv); library(MuMIn)
sei.gam0.0 <- gam(Total_unhealed ~ s(Calendar.date, bs="cs", k=5) + AgeClass + s(Depth_interval, bs="cs", k=5) + s(Length, bs="cs", k=5), data=sei.mod.dat, select=T, gamma=1.4, method="GCV.Cp", family=quasipoisson(link="log"), na.action="na.fail")
sei.gam0.1 <- update(sei.gam0.0, family=poisson(link="log"))
dred0 <- dredge(sei.gam0.1, rank="QAICc", chat=summary(sei.gam0.0)$scale)
# model with highest support
sei.gam0.6 <- update(sei.gam0.0, formula=Total_unhealed ~ s(Calendar.date, bs="cs", k=6) + AgeClass + Depth_interval)

## Plot

tiff("sei_gam0.6_smooths.tiff", width=1000, height=333, res=100)
cex.lab <- 2
cex.axis <- 2
par(mfrow=c(1,3), mar=c(5,5,3,2))
plot(sei.gam0.6, select=1, se=TRUE, seWithMean=TRUE, rug=TRUE, shade=TRUE, scale=0, cex.axis=cex.axis, cex.lab=cex.lab, ylab="s(Day of the year)", xlab="",
 trans=function(x){exp(x)}) # Poisson
mtext("Day of the year", side=1, line=3.5, cex=cex.lab*0.7)
myplot <- termplot(sei.gam0.6, terms="AgeClass", se=TRUE, ylab="Partial residuals", xlab="", xaxt="n", cex.lab=cex.lab, cex.axis=cex.axis, col.res=1, col.se=1, col.term=1)
labels <- levels(sei.gam0.6$model$AgeClass)
text(myplot, x=c(1:6), y=par("usr")[3]+par("usr")[3]*0.15, labels=labels, srt=45, adj=c(1.1,1.1), xpd=TRUE, cex=cex.axis*0.8)
axis(1, at=c(1:length(unique(sei.gam0.6$model$AgeClass))), labels=F)
mtext("Reproductive class", side=1, line=3.5, cex=cex.lab*0.7)
myplot <- termplot(sei.gam0.6, terms="Depth_interval", se=TRUE, ylab="Partials for depth", xlab="", xaxt="n", cex.lab=cex.lab, cex.axis=cex.axis, col.res=1, col.se=1, col.term=1)
axis(1, at=unique(sei.gam0.6$model$Depth_interval), labels=unique(sei.gam0.6$model$Depth_interval), cex.axis=cex.axis)
mtext("Depth interval", side=1, line=3.5, cex=cex.lab*0.7)
mtext("A", side=1, line=3, at=-10.5, cex=cex.lab-0.2)
dev.off()

# Fin whales

## Model

fin.gam0.0 <- gam(Total_unhealed ~ s(Calendar.date, bs="cs", k=6) + Sex + s(Depth_interval, bs="cs", k=3) + s(Length, bs="cs", k=4), data=fin.mod.dat, gamma=1.4, select=TRUE, family=quasipoisson(link="log"), na.action="na.fail")
fin.gam0.1 <- update(fin.gam0.0, family=poisson(link="log"))
summary(fin.gam0.0)
dred0 <- dredge(fin.gam0.1, rank="QAICc", chat=summary(fin.gam0.0)$scale)
# model with highest support
fin.gam0.3 <- update(fin.gam0.0, formula=Total_unhealed ~ s(Depth_interval, bs="cs", k=3) + s(Length, bs="cs", k=4) + s(Calendar.date, bs="cs", k=6))

## Plot

tiff("fin_gam03_smooths.tiff", width=1000, height=333, res=100)
cex.lab <- 2
cex.axis <- 2
par(mfrow=c(1,3), mar=c(5,5,3,2))
plot(fin.gam0.3, select=3, se=TRUE, seWithMean=TRUE, rug=TRUE, shade=TRUE, scale=0, cex.axis=cex.axis, cex.lab=cex.lab, ylab="s(Day of the year)", xlab="",
 trans=function(x){exp(x)}) # Poisson
mtext("Day of the year", side=1, line=3.5, cex=cex.lab*0.7)
plot(fin.gam0.3, select=2, se=TRUE, seWithMean=TRUE, rug=TRUE, shade=TRUE, scale=0, cex.axis=cex.axis, cex.lab=cex.lab, ylab="s(Length)", xlab="",
 trans=function(x){exp(x)}) # Poisson
mtext("Length (ft)", side=1, line=3.5, cex=cex.lab*0.7)
myplot <- plot(fin.gam0.3, select=1, se=TRUE, seWithMean=TRUE, rug=TRUE, shade=TRUE, scale=0, xaxt="n", cex.axis=cex.axis, cex.lab=cex.lab, ylab="s(Depth interval)", xlab="", trans=function(x){exp(x)}) # Poisson
axis(1, at=c(2:5), labels=c(2:5), cex.axis=cex.axis)
mtext("Depth interval", side=1, line=3.5, cex=cex.lab*0.7)
mtext("B", side=1, line=3, at=-7.4, cex=cex.lab-0.2)
dev.off()

# Offshore Bryde's whales

## Model

bryde.gam0.0 <- gam(Total_unhealed_Offshore ~ s(Calendar.date, bs="cs", k=5) + Sex + s(Depth_interval, bs="cs", k=3) + s(Length, bs="cs", k=4), data= bryde_offshore.sub, gamma=1.4, select=TRUE, family=quasipoisson(link="log"), na.action="na.fail")
bryde.gam0.1 <- update(bryde.gam0.0, family=poisson(link="log"))
dred0 <- dredge(bryde.gam0.1, rank="QAICc", chat=summary(bryde.gam0.0)$scale)
# model with highest support
bryde.gam0.2 <- update(bryde.gam0.0, formula=Total_unhealed_Offshore ~ s(Calendar.date, bs="cs", k=5) + s(Length, bs="cs", k=4) + Sex)

## Plot

tiff("bryde_gam0.2_smooths.tiff", width=1000, height=333, res=100)
cex.lab <- 2
cex.axis <- 2
par(mfrow=c(1,3), mar=c(5,5,3,2))
plot(bryde.gam0.2, select=1, se=TRUE, seWithMean=TRUE, rug=TRUE, shade=TRUE, scale=0, cex.axis=cex.axis, cex.lab=cex.lab, ylab="s(Day of the year)", xlab="",
 trans=function(x){exp(x)}) # Poisson
mtext("Day of the year", side=1, line=3.5, cex=cex.lab*0.7)
plot(bryde.gam0.2, select=2, se=TRUE, seWithMean=TRUE, rug=TRUE, shade=TRUE, scale=0, cex.axis=cex.axis, cex.lab=cex.lab, ylab="s(Length)", xlab="",
 trans=function(x){exp(x)}) # Poisson
mtext("Length (ft)", side=1, line=3.5, cex=cex.lab*0.7)
termplot(bryde.gam0.2, terms="Sex", se=TRUE, ylab="Partial residuals for sex", xlab="Sex", xaxt="n", cex.lab=cex.lab, cex.axis=cex.axis, col.res=1, col.se=1, col.term=1)
axis(1, at=c(1,2), labels=levels(bryde.gam0.2$model$Sex), cex.axis=cex.axis)
mtext("C", side=1, line=3, at=-5.75, cex=cex.lab-0.2)
dev.off()

# Sperm whales

## Model for OPTION A: missing data for total unhealed means NO RECENT SCARS

spermA.gam0.0 <- gam(Total_unhealedA ~ s(Calendar.date, bs="cs", k=5) + AgeClass + s(Depth_interval, bs="cs", k=3), data=sperm.subA, gamma=1.4, select=T, family=quasipoisson(link="log"), na.action="na.fail")
spermA.gam0.1 <- update(spermA.gam0.0, family=poisson(link="log"))
dredA0 <- dredge(spermA.gam0.1, rank="QAICc", chat=summary(spermA.gam0.0)$scale); dredA0
# model with highest support
spermA.gam0.2 <- update(spermA.gam0.0, formula=Total_unhealedA ~ s(Calendar.date, bs="cs", k=5) + AgeClass)

## Model for OPTION B: missing data for total unhealed means NO OBSERVATIONS

spermB.gam0.0 <- gam(Total_unhealedB ~ s(Calendar.date, bs="cs", k=5) + AgeClass + s(Depth_interval, bs="cs", k=3), data=sperm.subB, gamma=1.4, select=T, family=quasipoisson(link="log"), na.action="na.fail")
spermB.gam0.1 <- update(spermB.gam0.0, family=poisson(link="log"))
dredB0 <- dredge(spermB.gam0.1, rank="QAICc", chat=summary(spermB.gam0.0)$scale); dredB0
# model with highest support
spermB.gam0.2 <- update(spermB.gam0.0, formula=Total_unhealedA ~ s(Calendar.date, bs="cs", k=5) + AgeClass)

## Plot for OPTION A: missing data for total unhealed means NO RECENT SCARS

tiff("spermA.gam0.0_smooths.tiff", width=1000, height=333, res=100)
cex.lab <- cex.axis <- 2
par(mfrow=c(1,3), mar=c(5,5,2,1.5))
plot(spermA.gam0.0, select=1, se=TRUE, seWithMean=TRUE, rug=TRUE, shade=TRUE, scale=-1, cex.axis=cex.axis, cex.lab=cex.lab, ylab="s(Day of the year)", xlab="Day of the year",
 trans=function(x){exp(x)}) # Poisson
termplot(spermA.gam0.0, terms="AgeClass", se=TRUE, ylab="Partial residuals", xlab="Social grouping", xaxt="n", cex.lab=cex.lab, cex.axis=cex.axis, col.res=1, col.se=1, col.term=1)
axis(1, at=c(1:4), labels=levels(spermA.gam0.0$model$AgeClass), cex.axis=cex.axis)
plot(spermA.gam0.1, select=2, se=TRUE, seWithMean=TRUE, rug=TRUE, shade=TRUE, scale=-1, cex.axis=cex.axis, cex.lab=cex.lab, ylab="s(Depth interval)", xlab="Depth interval",
 trans=function(x){exp(x)}) # Poisson
mtext("A", side=1, line=3, at=-10.5, cex=cex.lab-0.2)
dev.off()

## Plot for OPTION B: missing data for total unhealed means NO OBSERVATIONS

tiff("spermB.gam0.1_smooths.tiff", width=1000, height=333, res=100)
cex.lab <- cex.axis <- 2
par(mfrow=c(1,3), mar=c(5,5,2,1.5))
plot(spermB.gam0.0, select=1, se=TRUE, seWithMean=TRUE, rug=TRUE, shade=TRUE, scale=-1, cex.axis=cex.axis, cex.lab=cex.lab, ylab="s(Day of the year)", xlab="Day of the year",
 trans=function(x){exp(x)}) # Poisson
termplot(spermB.gam0.0, terms="AgeClass", se=TRUE, ylab="Partial residuals", xlab="Social grouping", xaxt="n", cex.lab=cex.lab, cex.axis=cex.axis, col.res=1, col.se=1, col.term=1)
axis(1, at=c(1:4), labels=levels(spermB.gam0.0$model$AgeClass), cex.axis=cex.axis)
plot(spermB.gam0.0, select=2, se=TRUE, seWithMean=TRUE, rug=TRUE, shade=TRUE, scale=-1, cex.axis=cex.axis, cex.lab=cex.lab, ylab="s(Depth interval)", xlab="Depth interval",
 trans=function(x){exp(x)}) # Poisson
mtext("B", side=1, line=3, at=-10.5, cex=cex.lab-0.2)
dev.off()
