# Supplementary figures and images for: Identifying the “demon whale-biter”: Patterns of scarring on large whales attributed to a cookie-cutter shark Isistius sp
Source: PLoS One. 2016 Apr 7;11(4):e0152643. doi: 10.1371/journal.pone.0152643 (PMC4824425; doi:10.1371/journal.pone.0152643)

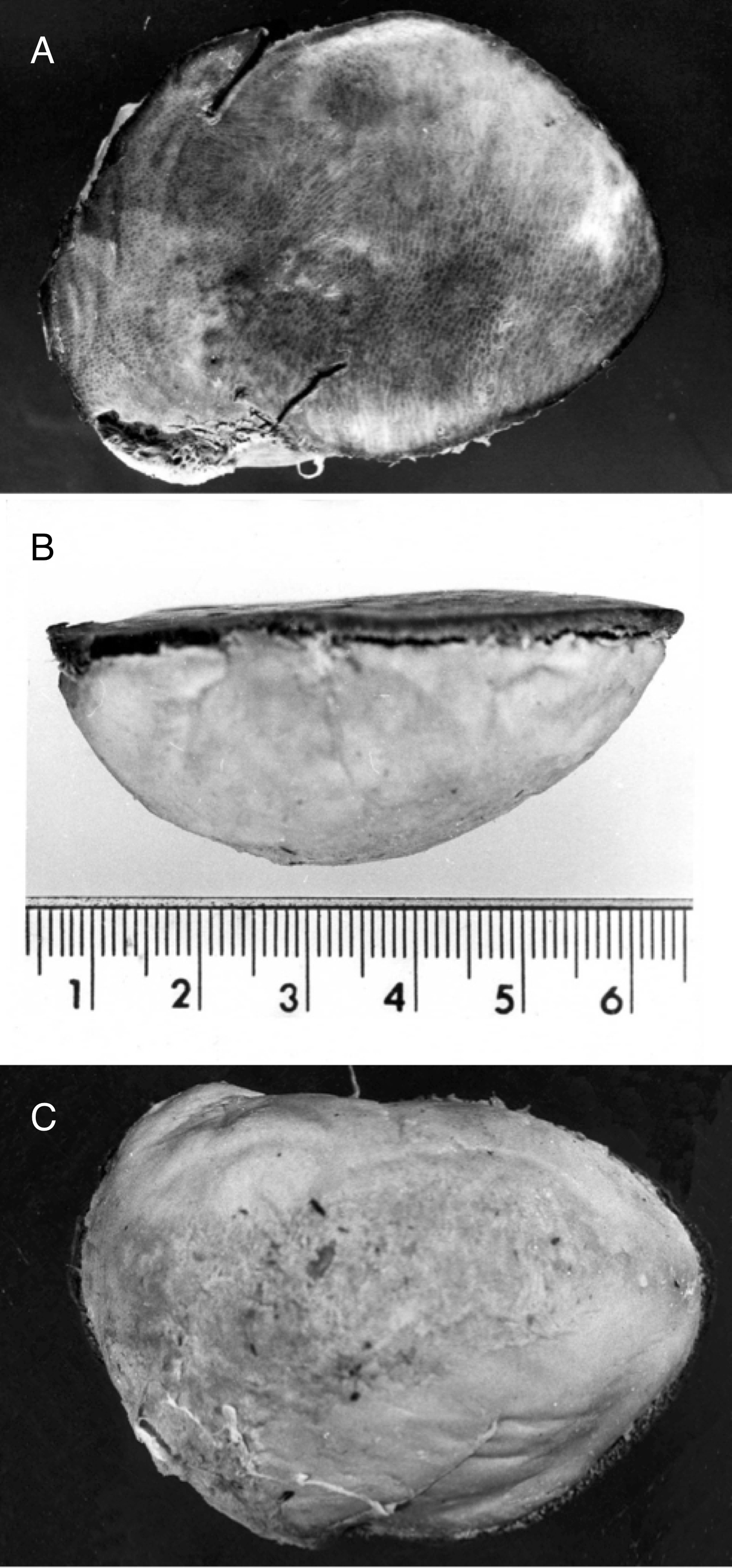

Supplement: S1 Fig — (TIF) [file pone.0152643.s003.tif]
