# Supplementary material for: Identifying the “demon whale-biter”: Patterns of scarring on large whales attributed to a cookie-cutter shark Isistius sp
Source: PLoS One. 2016 Apr 7;11(4):e0152643. doi: 10.1371/journal.pone.0152643 (PMC4824425; doi:10.1371/journal.pone.0152643)
Supplement: S1 Table — (DOCX) [file pone.0152643.s004.docx]

**S1 Table. Average numbers of unhealed bites on 226 mature sei whales of different reproductive classes examined at the Donkergat whaling station, South Africa, September/October 1963, with results of Tukey HSD Test.**

| Reproductive class | *n* | Mean number | SE | *P*<0.05 | *P*<0.01 |
| --- | --- | --- | --- | --- | --- |
| Primigravid females (PF) | 45 | 43.0 | 3.5 |  |  |
| Multigravid females (MF) | 99 | 41.6 | 2.2 |  |  |
| Lactating females (LF) | 6 | 83.5 | 15.0 |  | >PF, >MF, >MM |
| Resting females (RF) | 10 | 69.8 | 12.2 | >MF |  |
| Mature males (MM) | 66 | 51.4 | 4.2 |  |  |
