# Supplementary material for: Identifying the “demon whale-biter”: Patterns of scarring on large whales attributed to a cookie-cutter shark Isistius sp
Source: PLoS One. 2016 Apr 7;11(4):e0152643. doi: 10.1371/journal.pone.0152643 (PMC4824425; doi:10.1371/journal.pone.0152643)
Supplement: S1 Text — Including a comparison of wounds made by I. brasiliensis and I. plutodus, the biting technique of Isistius and a case study of a “reverse scoop” found in a sperm whale stomach and a review of other potential biting agents. (DOCX) [file pone.0152643.s005.docx]

# S1 Text. A review of the identity of the biting agent: evidence for and against Isistius as the biting agent

Although initially a great variety of causative agents for crater wounds seen on large whales was invoked (see Introduction, main text), more recently the list of candidates has narrowed to biting fish, specifically cookiecutter sharks of the genus *Isistius*. Because of the nominal rarity of *Isistius labialis* (only known from a single specimen from the East China Sea - [1]) and *I. plutodus* (about 10 records worldwide – [2], the much commoner *I. brasiliensis* has usually been considered by default as responsible for most of the crater wounds seen. This species occurs in water depths of 200 – 3,700 m [3], but is caught at shallower depths, particularly in surface trawls at night, leading to the hypothesis that it performs diel vertical migrations [4,5]. It is presumably during these visits to surface waters at night that most of the attacks on whales and other large vertebrates occur [6].

Direct observations of attacks leading to crater wounds and the subsequent identification of the attacker are obviously difficult to obtain. The only direct evidence, in fact, of *I. brasiliensis* being responsible for crater-like wounds is the simultaneous capture of one with a fish that it was biting [7]. It is also possible that more than one species of *Isistius* is involved in the case of cetaceans [8]. Examination of stomach contents of a single *I. plutodus* included a plug of fish muscle measuring ca 70 x 36 mm: 10% of 60 stomachs of *I. brasiliensis* examined with food contained pieces of cetacean skin and blubber, dimensions not given [9].

## A comparison of wounds made by *I. brasiliensis* and *I. plutodus*

Some authors have claimed that the bites of *I. brasiliensis* and *plutodus* can be distinguished by their size and shape, with *brasiliensis* making circular bitemarks and *plutodus* oval bitemarks [10–12], although the former commented that *plutodus* can produce both circular and elongated wounds. This distinction seems to have arisen from the hypothesis that *brasiliensis* makes its predatory approach from in front of its target and then uses hydrodynamic drag to rotate its body through 180° [10,13], which logically should then produce a fairly symmetrical plug of flesh (the assumption being that presumably *plutodus* with its larger dentition does not employ this tactic). The authors of [14] dispute the involvement of hydrodynamic drag as *Isistius* cuts similar plugs from carcasses, and propose that any rotation of the body is spontaneous rather than induced. The orientation of most half-scoops, however, argues against the hypothesis of a frontal approach, at least so far as those on baleen whales are concerned. If the attack came from ahead of the whale, then an incomplete bite by the powerful lower jaw would leave a C-shaped wound with the incomplete section facing aft. In practice, however, such half-scoops are almost invariably facing forward, indicating an attack from behind the whale [15]. An approach from behind (and below) would also seem more consistent with the concentration of bitemarks on the flanks and belly of large cetaceans.

A 400 mm female *I. brasiliensis* caught in a midwater trawl punched holes in the cod end that were both circular and elliptical in shape (Fig 1, main text). Three of the holes had a longest diameter of 40 mm and two 28 mm: a 6th hole on the other side of the sleeve was also 40 mm in diameter. The captured shark had a maximum mandibular width of 37 mm, but from the 2 sizes of holes it is possible a second, smaller shark was present that escaped through one of the holes made by the larger one (Jon Staiger, in litt 10 May 1972). Another *I. brasiliensis* taken in a midwater trawl and measuring about 274 mm had a distinctly elliptical plug of fish tissue in the stomach (Fig 1, main text). To date there has been no demonstration of different-sized plugs of cetacean skin or blubber in the stomachs of *I. plutodus* or *I. brasiliensis*, but there is the recognition that the size of the wounds may be partly dependent on the size class/sex of the shark [5].

## The biting technique of *Isistius*: a case study of a “reverse scoop” found in a sperm whale stomach

On 2 September 1963 PBB collected what was clearly the reverse “scoop” of an oval bite, from the forestomach of a 32 ft female sperm whale landed at the Donkergat whaling station (S1 Fig). The scoop had a maximum length and width of 58 and 41mm respectively, and was 23.5 mm deep at its maximum: after fixation it weighed 70 g. It is now lodged in the Iziko South African Museum in Cape Town. The whale was killed about 218 km offshore from the whaling station, in a water depth of about 3,000 m. The remainder of the forestomach contents consisted of 4 mysids (probably *Gnathophausia ingens*), remains resembling those from ceratid angler fish, a few cephalopod beaks and digested squid remains. In the second (pyloric) stomach there were a few cephalopod beaks, nematodes and mysid remains. As these items did not include any organism that could have been responsible for the bite, it can only be assumed that the biting agent disgorged the scoop of blubber which was then swallowed (presumably accidentally) by the whale.

The shape and dimensions of, and markings on the scoop provide important evidence of the technique used by the biter and clues to its identity. The scoop is roughly elliptical in dorsal view and in lateral profile is smoothly convex ventrally, albeit with a deepest point slightly closer to one end (0.44 of total length) than the other: the impression is gained of one continuous bite. Grooves on the ventral surface suggest that the biting action was along the longer axis of the scoop rather than across it. The more symmetrical and cleaner edge of the righthand end of the scoop in S1 Fig image A suggests that this represents the initial strike by the lower jaw, while a series of small pin-prick incisions running in a semi-circle near the opposite end of the scoop (and connecting with partial cuts on either side of the scoop) indicate the likely site of attachment of the upper teeth. If this is correct, it suggests the initial “bite” at impact measured 42 x 44 mm, and was roughly circular. The final scoop, however, measured 42 x 58 mm, or distinctly oval in shape.

Close examination of the roughly U-shaped righthand end of the scoop in S1 Fig image A reveals 12-13 tiny but evenly-spaced indentations in the outline of the one side of the “U”: if these are in fact impressions of individual teeth it suggests a total mandibular tooth count of 24-26 (cf counts of 25-31 and 19 for *I. brasiliensis* and *I. plutodus* respectively [4]).

An anatomical study of *I. brasiliensis* revealed several features associated with the head and jaws that distinguished it from other squaloid sharks [14]. Among these are 1) the upper teeth are wholly inside the lower teeth when the mouth is closed (i.e. the 2 rows fail to appose each other), and 2) the palatoquadrate (bearing the upper teeth) is subdivided into an anterior and posterior section, with some dorsal warp possible anteriorly. These morphological features suggested to the authors of [14] a means of enlarging the bite, the upper jaw flexing at the central articulation on impact, effectively making the oral area greater, while the upper teeth anchored the shark to its prey. The consequences of this must be that the shark’s normal underbite becomes exaggerated, creating an elliptical rather than circular scoop. The flexibility of the biting apparatus might also account for the variation in bite shape, with the degree of elongation of the wound being dependent on the resilience of the prey’s integument and possibly the vigour of the attack.

## Other potential biting agents

One objection to an *Isistius* species being responsible for the fresh bites in South African waters is that the genus is known from very few records from the area [16]. The southern limit to its distribution in the South Atlantic is poorly known but has been described as 34-35°S [17] while specimen records (some poorly documented) extend to about 37°S (Table A). Although the finding of an undigested scoop in the forestomach of a sperm whale (S1 Fig) must indicate that the biting agent occurs very close to if not within the Saldanha Bay whaling ground, there are no *Isistius* specimens in South African museums with a South African provenance (Roger Bills, M. Bougaardt, M. Smale, pers. comm.). There appear to be only 5 specimen records of *I. brasiliensis* from south of 30°S and between 0 and 40°E, or in the vicinity of South Africa (Table A). Two of these were taken in midwater research trawls about 935 km and 1,025 km off the west coast of South Africa [18]. The authors of (1987) [9] illustrate a third record at about 36°S 23°E, or 220 km offshore on the eastern slope of the Agulhas Bank, but attempts to verify this latter record by locating the specimen have so far proved unsuccessful (although it is a reasonable assumption that it originated from the Dana Expedition that was sampling south of South Africa early in 1930). Two other records are illustrated on some distribution maps (e.g. www.aquamaps.org, version of Aug. 2013. Web. Accessed 9 Oct. 2014) as occurring in the half-degree cells 34.5-35°S 20-20.5°E and 37-37.5°S 21-21.5°E, but so far the sources of these records have not been established: from its proximity it is possible that the latter location may refer to the record mentioned above [9].

Table A. Records of *Isistius* sp off South Africa

| Date | Latitude (°S) | Longitude (°E) | No. | Source | Provenance |
| --- | --- | --- | --- | --- | --- |
| 30-Mar-71 | 33 | 07.83 | 1 | Krefft (1980) | MT 1600 |
| 31-Mar-71 | 30.15 | 05.43 | 1 | Krefft (1980) | MT 1600 |
| Perhaps 1930 | ~36 | ~23 | 1 | Jahn & Haedrich (1987) | Dana collection? |
| 25-Nov-02 | 30.1167 | 14.56667 | 1 | DAFF observer | Longline bycatch |
| 19-May-03 | 27.8688 | 10.04167 | 2 | DAFF observer | Longline bycatch |
| 10-Apr-03 | 31.8142 | 31.67433 | 16 | DAFF observer | Longline bycatch |
| 23-Jun-07 | 34.9693 | 24.66833 | 1 | DAFF observer | Longline bycatch |
| 26-Jun-07 | 33.5268 | 28.34067 | 2 | DAFF observer | Longline bycatch |
| 08-Sep-07 | 31.6197 | 30.43617 | 1 | DAFF observer | Longline bycatch |
| 05-Aug-08 | 37.04 | 23.26667 | 1 | DAFF observer | Longline bycatch |

The species has also has been nominally recorded as bycatch in the tuna/swordfish longline fishery centred on and around the Agulhas Bank (between 25° and 40°S and between 5° and 50°E), where observer data indicated it formed 0.1% by number of the observed bycatch with a catch rate of about 2 every 100,000 hooks in the South African fishery [19]. Data from the most reliable period of monitoring (2002-2008) include 7 such records, all but one involving the take of 1-2 fish, the exception being a catch of 16 individuals: catches were made between 27° and 37°S, but 5 between 30° and 35°S (Table 1). No voucher material (specimens or images) appears to exist for these records.

The possible involvement of the somniosid shark *Centroscymnus coelolepis* as an ectoparasite of cetaceans originated with the findings of [20]. The contents of 3 out of 12 *C. coelolepsis* stomachs examined contained cetacean remains, characterized as “1 piece cetacean skin, … several pieces of muscle”, “2 pieces cetacean blubber” and “2 pieces of odontocete muscle” respectively. Characteristics of the blubber and muscle suggested that they came from sperm whales. While the presence of muscle implied a dead whale was involved, the skin and blubber suggested that a living whale might have been attacked during a feeding dive (the fish having been caught at depths of 998 – 1,975 m). The size of the blubber plugs (38 x 33 x 15, 23 x 23 x 9 mm) however are more circular and shallower than most of the wounds seen on whales at Donkergat (and the scoop recovered from a sperm whale stomach). The other source sometimes cited (e.g. by [21]) as implicating *C. coelolepsis* in attacks on living whales also involved the discovery of whale flesh alone or along with blubber in 6 stomachs [22], despite the authors concluding that the whale remains were probably the result of scavenging. However, as *C. coelolepsis* is found at depths of 150-3,700 m, and usually deeper than 600 m [3], the possibility of the shark encountering any living large whale (apart from a sperm whale) would seem remote.

The proposal that lampreys were responsible for some of the wounds on large whales seems to have originated with Japha (1910 – in [23]), who described round wounds about 50mm in diameter on sei and fin whales in the eastern North Atlantic. This hypothesis was revived by [24], who ascribed all crater wounds on fin, humpback, sei and sperm whales off Vancouver Island in the eastern North Pacific to the bites of the lamprey *Entosphenus tridentatus*, even though there were clearly 2 types of bitemarks involved, as [25] found when describing bitemarks on whales in the western North Pacific, Japan and the Okhotsk Sea. The bitemarks described here, attributable to *Isistius*, are distinct from those produced by lampreys. [25] found that wounds that could be attributed to lampreys were circular, oval, horse-shoe shaped or belt-like, a maximum of 10mm deep and marks left by the teeth on the sucking disc were visible: wounds inflicted by another source were usually oval, much deeper and bore no internal tooth marks. [26] illustrated wounds on the tail flukes of unidentified cetacean species stranded in New Zealand that he attributed to the bites of lampreys, but the majority of these were incomplete C-shaped bites typical of *Isistius*. Although he stated that a lamprey was still attached to one elliptical wound, this appears to be a partly healed scar. More recently, the involvement of lampreys in biting large whales has been confirmed through sightings of the fish attached to right whales [23], minke whales [27,28] and killer whales [29]. All records occurred in the North Atlantic, and all involved the sea lamprey *Petromyzon marinus*. Concomitant observations of bite wounds confirmed the characteristics described by [25], and indicated that the resultant scars were not permanent, disappearing within a year [28,29].

No lamprey-like bitemarks or scars were recorded on whales examined at Donkergat in 1963, although in the early years of whaling at Saldanha Bay whalers reported to both [30] and [31] that hagfish (myxinoid) made wounds on both Bryde’s and blue whales, but they dropped off when the whale was killed or before it was pulled from the water, so no specimens were ever examined. [30] speculated on whether the fish was really scavenging on dead whales rather than attacking live ones. Neither sets of authors mentioned whalesuckers (*Remora australis*) that were seen on live (and even some dead) blue whales at Donkergat in the 1960s, so it is possible that some of these records may be misidentifications.

## References

1. Meng Q, Zhu Y, Li S. A new species of Dalatiidae (*Squaliformes*) of China. Acta Zootaxon Sin. 1985;4: 442–444.

2. Kyne, P.M., Gerber, L, Sherrill-Mix SA. *Isistius plutodus*. In: UCN 2013. IUCN Red List of Threatened Species. [Internet]. 2006 [cited 13 Nov 2013]. Available: www.iucnredlist.org

3. Kiraly S, Moore J, Jasinski P. Deepwater and other sharks of the US Atlantic ocean exclusive economic zone. Mar Fish Rev. 2003;65: 1–63.

4. Compagno L. FAO Species Catalogue. Vol. 4. Sharks of the world. An annotated and illustrated catalogue of shark species known to date. Part 1: Hexanchiformes to Lamniformes. FAO Fish. Synop. 1984.

5. Papastamatiou YP, Wetherbee BM, O’Sullivan J, Goodmanlowe GD, Lowe CG. Foraging ecology of Cookiecutter Sharks (*Isistius brasiliensis*) on pelagic fishes in Hawaii, inferred from prey bite wounds. Environ Biol Fishes. 2010;88: 361–368.

6. Hoyos-Padilla M, Papastamatiou YP, O’Sullivan J, Lowe CG. Observation of an attack by a cookiecutter shark (*Isistius brasiliensis*) on a white shark (*Carcharodon carcharias*). Pacific Sci. University of Hawai’i Press; 2013;67: 129–134. doi:10.2984/67.1.10

7. Makino Y, Tachihara, K, Ageda S, Arao T, Fuke C MT. Peculiar circular and C-shaped injuries on a body from the sea. Am J Forensic Med Pathol. 2004;25: 169–171.

8. Dwyer S, Visser I. Cookie cutter shark (*Isistius* sp.) bites on cetaceans, with particular reference to killer whales (*Orca*) (*Orcinus orca*). Aquat Mamm. 2011;37: 111–138.

9. Jahn A, Haedrich R. Notes on the pelagic squaloid shark *Isistius brasiliensis*. Biol Oceanogr. 1988;5: 297–309.

10. Williams Jr E, Bunkley-Williams L. Parasites of offshore big game fishes of Puerto Rico and the western Atlantic. Puerto Rico Department of Natural and Environmental Resources, San Juan, PR, and the University of Puerto Rico; 1996.

11. Pérez-Zayas, J.J., A.A. Mignucci-Giannoni, G.M. Toyos-González, R.J. Rosario-Delestre EHWJ. Incidental predation by a largetooth cookiecutter shark on a Cuvier’s beaked whale in Puerto Rico. Aquat Mamm. 2002;28: 308–311.

12. Souto, L.R.A., J.G.A. Oliveira, J. de A.C. da C. Nunes, R. Maia-Nogueira CLSS. Análise das mordidas de tubarões-charuto, *Isistius* spp. (*Squaliformes: Dalatiidae*) em cetáceos (Mammalia: Cetacea) no litoral da Bahia, Nordeste do Brasil. Biotemas. 2007;20: 19–25.

13. Jones EC. *Isistius brasiliensis* a squalid shark, the probable cause of wounds on fishes and cetaceans. Fish Bull US. 1971;69: 791–798.

14. Shirai S, Nakaya K. Functional morphology of feeding apparatus of the cookie-cutter shark, *Isistius brasiliensis* (*Elasmobranchii, Dalatiinae*). Zoolog Sci. 1992;9: 811–821.

15. Best PB. Whales and dolphins of the southern African subregion. Cape Town: Cambridge University Press; 2007.

16. Compagno L. An overview of chondrichthyan systematics and biodiversity in southern Africa. Trans R Soc South Africa. 1999;54: 75–120.

17. Shevchenko V. Application of white scars to the study of the location and migrations of sei whale populations in Area III of the Antarctic. Rep Int Whal Comm. 1977;Special Is: 130–134.

18. Krefft G. Results of the research cruises of FRV ”Walther Herwig“ to South America. LIII. Sharks from the pelagic trawl catches obtained during Atlantic transects, including some specimens from other cruises. Arch für Fischereiwiss. 1980;30: 1–16.

19. Petersen SL, Honig MB, Ryan PG, Underhill LG, Compagno LJ. Pelagic shark bycatch in the tuna- and swordfish-directed longline fishery off southern Africa. African J Mar Sci. 2009;31: 215–225.

20. Clarke MR, Merrett N. The Significance of Squid, Whale and Other Remains From the Stomachs of Bottom-Living Deep-Sea Fish. J Mar Biol Assoc United Kingdom. Cambridge University Press; 1972;52: 599–603.

21. Heithaus M. Shark attacks on bottlenose dolphins (*Tursiops aduncus*) in Shark Bay, Western Australia: attack rate, bite scar frequencies, and attack seasonality. Mar Mammal Sci. 2001;17: 526–539.

22. Mauchline J, Gordon JDM. Diets of the sharks and chimaeroids of the Rockall Trough, northeastern Atlantic Ocean. Mar Biol. 1983;75: 269–278.

23. Nichols OC, Hamilton PK. Occurrence of the parasitic sea lamprey *Petromyzon marinus* on western North Atlantic right whales *Eubalaena glacialis*. Environ Biol Fishes. 2004;71: 413–417.

24. Pike G. Lamprey marks on whales. J Fish Board Canada. 1951;8: 275–280.

25. Nemoto T. White scars on whales (I) Lamprey marks. Sci Reports Whales Res Institute, Tokyo. 1955;10: 69–77.

26. Robson F. Strandings: Ways to Save Whales: a Humane Conservationist’s Guide. Johannesburg: The Science Press; 1984.

27. Nichols OC, Tscherter UT. Feeding of sea lampreys Petromyzon marinus on minke whales *Balaenoptera acutorostrata* in the St Lawrence Estuary, Canada. J Fish Biol. 2011;78: 338–343.

28. Olafsdóttir D, Shinn AP. Epibiotic macrofauna on common minke whales, *Balaenoptera acutorostrata* Lacépède, 1804, in Icelandic waters. Parasites and Vectors. 2013;6: 1–10. doi:10.1186/1756-3305-6-105

29. Samarra FIP, Fennell A, Aoki K, Deecke VB, Miller PJO. Persistence of skin marks on killer whales (*Orcinus orca*) caused by the parasitic sea lamprey (*Petromyzon marinus*) in Iceland. Mar Mammal Sci. 2012;28: 395–401.

30. Olsen Ø. Hvaler og hvalfangst i Sydafrika. Bergen Museums Aarb. 1914;1914-15: 1–56.

31. Mackintosh N, Wheeler J. Southern blue and fin whales. Discov Reports. 1929;1: 257–540.
