# Supplementary material for: Identifying the “demon whale-biter”: Patterns of scarring on large whales attributed to a cookie-cutter shark Isistius sp
Source: PLoS One. 2016 Apr 7;11(4):e0152643. doi: 10.1371/journal.pone.0152643 (PMC4824425; doi:10.1371/journal.pone.0152643)
Supplement: S2 Code — (DOCX) [file pone.0152643.s006.docx]

S2 code. Code for fitting GAMs to the proportion of unhealed bitemarks that were recent

Models for the proportion of unhealed bitemarks that were recent, were fitted with a quasibinomial error structure and a logit link function. Where non-linear relationships between the response and explanatory variables were present, these terms were fitted as smooth functions using the “cs” basis function with shrinkage [1]. This allows the effect of covariates to be shrunk to zero if there is no relationship with the response. Below is the code used to model the proportion of unhealed bitemarks that were recent in each whale species and the code used to produce the plots of the fitted relationships for the models with highest support. The number of knots was checked following routines documented in the mgcv library [2].

# Sei whales

## Models

library(mgcv); library(MuMIn)
sei.prop.gam0.0b <- gam(Proportion_Recent ~ s(Calendar.date, bs="cs", k=5) + AgeClass + Depth_interval + s(Length, bs="cs", k=3), data=sei.sub, family=quasibinomial(link="logit"), weights=Total_unhealed, na.action="na.fail")
sei.prop.gam0.1b <- update(sei.prop.gam0.0b, family=binomial(link="logit"))
dred.prop0b <- dredge(sei.prop.gam0.1b, rank="QAICc", chat=summary(sei.prop.gam0.0b)$dispersion)
# model including reproductive class
sei.prop.gam0.2b <- update(sei.prop.gam0.0b, formula=Proportion_Recent ~ AgeClass + s(Calendar.date, bs="cs", k=5) + Depth_interval)
# model including length
sei.prop.gam0.3b <- update(sei.prop.gam0.0b, formula=Proportion_Recent ~ s(Calendar.date, bs="cs", k=5) + Depth_interval + s(Length, bs="cs", k=3))

## Plot for model including reproductive class

tiff("sei_PROPgam0.2b_smooths.tiff", width=1000, height=333, res=100)
cex.lab <- cex.axis <- 2
par(mfrow=c(1,3), mar=c(5,5,3,2))
cex.lab <- 2
cex.axis <- 2
plot(sei.prop.gam0.2b, select=1, se=TRUE, seWithMean=TRUE, rug=TRUE, shade=TRUE, scale=0, cex.axis=cex.axis, cex.lab=cex.lab, ylab="s(Day of the year)", xlab="",
 trans=plogis) # binomial
mtext("Day of the year", side=1, line=3.5, cex=cex.lab*0.7)
myplot <- termplot(sei.prop.gam0.2b, terms="AgeClass", se=TRUE, ylab="Partial residuals", xlab="", xaxt="n", cex.lab=cex.lab, cex.axis=cex.axis, col.res=1, col.se=1, col.term=1)
labels <- levels(sei.prop.gam0.2b$model$AgeClass)
text(myplot, x=c(1:6), y=par("usr")[3]+par("usr")[3]*0.05, labels=labels, srt=45, adj=c(1.1,1.1), xpd=TRUE, cex=cex.axis*0.8)
axis(1, at=c(1:length(unique(sei.prop.gam0.2b$model$AgeClass))), labels=F)
mtext("Reproductive class", side=1, line=3.5, cex=cex.lab*0.7)
myplot <- termplot(sei.prop.gam0.2b, terms="Depth_interval", se=TRUE, ylab="Partials for depth", xlab="", xaxt="n", cex.lab=cex.lab, cex.axis=cex.axis, col.res=1, col.se=1, col.term=1)
axis(1, at=unique(sei.prop.gam0.2b$model$Depth_interval), labels=unique(sei.prop.gam0.2b$model$Depth_interval), cex.axis=cex.axis)
mtext("Depth interval", side=1, line=3.5, cex=cex.lab*0.7)
mtext("A", side=1, line=3, at=-10.5, cex=cex.lab-0.2)
dev.off()

## Plot for model including length

tiff("sei_PROPgam0.3b_smooths.tiff", width=1000, height=333, res=100)
cex.lab <- cex.axis <- 2
par(mfrow=c(1,3), mar=c(5,5,3,2))
plot(sei.prop.gam0.3b, select=1, se=TRUE, seWithMean=TRUE, rug=TRUE, shade=TRUE, scale=0, cex.axis=cex.axis, cex.lab=cex.lab, ylab="s(Day of the year)", xlab="",
 trans=plogis) # binomial
mtext("Day of the year", side=1, line=3.5, cex=cex.lab*0.7)
plot(sei.prop.gam0.3b, select=2, se=TRUE, seWithMean=TRUE, rug=TRUE, shade=TRUE, scale=0, cex.axis=cex.axis, cex.lab=cex.lab, ylab="s(Length)", xlab="", trans=plogis)
mtext("Length (ft)", side=1, line=3.5, cex=cex.lab*0.7)
myplot <- termplot(sei.prop.gam0.3b, terms="Depth_interval", se=TRUE, ylab="Partials for depth", xlab="", xaxt="n", cex.lab=cex.lab, cex.axis=cex.axis, col.res=1, col.se=1, col.term=1)
axis(1, at=unique(sei.prop.gam0.3b$model$Depth_interval), labels=unique(sei.prop.gam0.3b$model$Depth_interval), cex.axis=cex.axis)
mtext("Depth interval", side=1, line=3.5, cex=cex.lab*0.7)
mtext("B", side=1, line=3, at=-10.5, cex=cex.lab-0.2)
dev.off()

# Fin whales

## Model

library(mgcv); library(MuMIn)
fin.prop.gam0.0a <- gam(Proportion_Recent ~ Calendar.date + Sex + s(Depth_interval, bs="cs", k=3) + Length, data=fin.sub, family=quasibinomial(link="logit"), weights=Total_unhealed, na.action="na.fail")
fin.prop.gam0.1a <- update(fin.prop.gam0.0a, family=binomial(link="logit"))
dredf.prop0a <- dredge(fin.prop.gam0.1a, rank="QAICc", chat=summary(fin.prop.gam0.0a)$dispersion)
fin.prop.gam0.2a <- update(fin.prop.gam0.0a, formula=Proportion_Recent ~ s(Calendar.date, bs="cs", k=4))

The models including day of the year and day of the year plus depth were practically identical in terms of QAICc and hence weight. The term for depth had a flat line (no relationship with the response) as a fitted relationship so we chose not to consider the model including it.

## Plot

tiff("fin_PROPgam0.2a_smooths.tiff", width=1000, height=333, res=100)
cex.lab <- cex.axis <- 2
par(mfrow=c(1,3), mar=c(5,5,3,2))
# fin.prop.gam1
plot(fin.prop.gam0.2a, select=1, se=TRUE, seWithMean=TRUE, rug=TRUE, shade=TRUE, scale=-1, cex.axis=cex.axis, cex.lab=cex.lab, ylab="s(Day of the year)", xlab="Day of the year",
 trans=plogis) # binomial
mtext("C", side=1, line=3, at=110, cex=cex.lab-0.2)
dev.off()
