# Supplementary material for: Identifying the “demon whale-biter”: Patterns of scarring on large whales attributed to a cookie-cutter shark Isistius sp
Source: PLoS One. 2016 Apr 7;11(4):e0152643. doi: 10.1371/journal.pone.0152643 (PMC4824425; doi:10.1371/journal.pone.0152643)
Supplement: S2 Table — (DOCX) [file pone.0152643.s007.docx]

**S2 Table. Location of unhealed bitemarks on 169 sperm whales examined at the Donkergat whaling station, South Africa, 1963**

| Location | Number of bitemarks | | | | % of total |
| --- | --- | --- | --- | --- | --- |
|  | Fresh | Freshish | Brown | Total |  |
| Front of head | 1 |  | 18 | 19 | 7.5 |
| Side of head | 7 | 1 | 46 | 54 | 21.3 |
| Top of head | 1 | 1 | 12 | 14 | 5.5 |
| Head (unspecified) |  |  | 6 | 6 | 2.4 |
| Chest | 1 | 1 | 20 | 22 | 8.7 |
| Neck/Shoulder |  |  | 5 | 5 | 2.0 |
| Flipper | 1 |  | 2 | 3 | 1.2 |
| Belly | 1 |  | 4 | 5 | 2.0 |
| Genital region | 1 |  | 19 | 20 | 7.9 |
| Flank/side | 6 | 2 | 25 | 33 | 13.0 |
| Back | 4 | 2 | 26 | 32 | 12.6 |
| Peduncle | 2 | 2 | 29 | 33 | 13.0 |
| Tail | 1 |  | 6 | 7 | 2.8 |
| Total | 26 | 9 | 218 | 253 |  |
