# Supplementary material for: Identifying the “demon whale-biter”: Patterns of scarring on large whales attributed to a cookie-cutter shark Isistius sp
Source: PLoS One. 2016 Apr 7;11(4):e0152643. doi: 10.1371/journal.pone.0152643 (PMC4824425; doi:10.1371/journal.pone.0152643)
Supplement: S2 Text — (DOCX) [file pone.0152643.s008.docx]

**S2 Text. Interpretation of missing records**

Some uncertainty exists over entries in the database that could be interpreted as either absence or a failure to record presence/absence. Thus for instance in some whales the presence or absence of recent bitemarks would be specifically noted, while in others there was simply no mention of any recent bitemarks. In other whales there might be no entries for healed scars but data recorded on the presence/absence of unhealed bitemarks. At this juncture it is difficult to recollect the circumstances surrounding all such entries, but we believe the following is an appropriate interpretation of such records for baleen whales.

1. It would be biologically unlikely for there to be unhealed bitemarks present but no healed scars, so if the presence/absence of unhealed bitemarks is recorded but with no mention of healed scars, we would rate this as “no observation” for healed scars.
2. If the presence/absence of healed scars has been recorded and classified, then the assumption is made that any unhealed bitemarks would also have been recorded if present. Hence if under these circumstances there is no mention of unhealed bitemarks then this is considered a genuine instance of “no recent bitemarks”.
3. If there is no mention of either healed or unhealed scarring then these are considered as instances of “no observation” for both categories.

In the case of sperm whales, where healed scars were not recorded, the situation is not so straightforward, and we have had to proceed with 2 alternative interpretations, namely, where entries fail to specifically record presence/absence of unhealed bitemarks we assume it was a case of either (Option A) “no recent bitemarks” or (Option B) “no observation”.
