# Supplementary material for: Identifying the “demon whale-biter”: Patterns of scarring on large whales attributed to a cookie-cutter shark Isistius sp
Source: PLoS One. 2016 Apr 7;11(4):e0152643. doi: 10.1371/journal.pone.0152643 (PMC4824425; doi:10.1371/journal.pone.0152643)
Supplement: S3 Table — (DOCX) [file pone.0152643.s009.docx]

**S3 Table. Location of unhealed incomplete bites on whales examined at the Donkergat whaling station, South Africa, 1963**

| Location | Number of half scoops | | | |
| --- | --- | --- | --- | --- |
|  | Sperm | Sei | Fin | Bryde |
| Front of head | 3 |  |  |  |
| Side of head/mandible | 1 | 2 | 1 |  |
| Top of head | 1 | 1 |  |  |
| Head (unspecified) | 2 |  |  |  |
| Chest | 2 |  |  |  |
| Neck/Shoulder |  |  |  |  |
| Flipper |  |  |  |  |
| Belly |  |  |  |  |
| Genital region | 1 |  |  |  |
| Flank/side | 2 |  |  |  |
| Back/Dorsal fin | 2 | 1 |  |  |
| Peduncle | 1 | 1 |  |  |
| Tail | 1 | 10 |  | 1 |
