# Supplementary material for: Identifying the “demon whale-biter”: Patterns of scarring on large whales attributed to a cookie-cutter shark Isistius sp
Source: PLoS One. 2016 Apr 7;11(4):e0152643. doi: 10.1371/journal.pone.0152643 (PMC4824425; doi:10.1371/journal.pone.0152643)
Supplement: S3 Text — (DOCX) [file pone.0152643.s010.docx]

**S3 Text. Account of an outlying observation of a sperm whale with a large number of unhealed bitemarks**

Excluded from these figures is an apparent outlier. A 32 ft female sperm whale landed on 16 September 1963 was recorded with 74 healing wounds, or more than 4 times the next highest total (17). This female had a scrolled lower jaw, bent to the right through 180^o^, with all but the posterior 7 teeth missing on the left side (and the first tooth on the right side). It is possible that this female was incapacitated in some way, and so more vulnerable to attack by *Isistius* (as suggested by [1]). However such deformities occur in all sperm whale populations examined to date, with an incidence of 0.6% off the west coast of South Africa [2]. There were 4 other whales with mandibles bent through 45-90° examined at Donkergat in 1963, all males 34-42 ft long, and the number of recent bitemarks recorded in 3 of them ranged from 0 to 2, with an average of 1.3. Hence the large number of wounds on the female may not be related to its deformed mandible.

**References**

1. Gasparini J, Sazima I. A stranded melon‐headed whale, *Peponocephala electra*, in Southeastern Brazil, with comments on wounds from the cookiecutter shark, *Isistius brasiliensis*. Mar Mammal Sci. 1996;12: 308–312.

2. Best, P.B, Gambell R. A comparison of the external characters of sperm whales off South Africa. Nor Hvalfangst-tidende. 1968;57: 146–164.
