# Supplementary material for: Identifying the “demon whale-biter”: Patterns of scarring on large whales attributed to a cookie-cutter shark Isistius sp
Source: PLoS One. 2016 Apr 7;11(4):e0152643. doi: 10.1371/journal.pone.0152643 (PMC4824425; doi:10.1371/journal.pone.0152643)
Supplement: S4 Table — (DOCX) [file pone.0152643.s011.docx]

**S4 Table. Incidence of recent bites on large whales landed at the Donkergat whaling station, South Africa, 1963, and mean number of bites per whale bitten, by depth interval**

| Depth interval (m) | *n* | % bites present | Mean no. of bites ± SE (*n*) | Tukey HSD Test | |
| --- | --- | --- | --- | --- | --- |
|  |  |  |  | *P*< 0.05 | *P*<0.01 |
| 0 - 200 | 19 | 0 |  |  |  |
| 200 – 1,000 | 191 | 38.2 | 1.8 ± 0.2 (73) | <3,000 m + |  |
| 1,000 – 2,000 | 188 | 58.5 | 1.6 ± 0.1 (110) |  | <3,000 m + |
| 2,000 – 3,000 | 227 | 70.9 | 1.8 ± 0.1 (161) | <3,000 m + |  |
| 3,000 + | 63 | 82.5 | 2.4 ± 0.3 (52) |  |  |
